# Dystrophin Deficiency Leads to Genomic Instability in Human Pluripotent Stem Cells via NO Synthase-Induced Oxidative Stress

**DOI:** 10.3390/cells8010053

**Published:** 2019-01-15

**Authors:** Sarka Jelinkova, Petr Fojtik, Aneta Kohutova, Aleksandra Vilotic, Lenka Marková, Martin Pesl, Tereza Jurakova, Miriama Kruta, Jan Vrbsky, Renata Gaillyova, Iveta Valášková, Ivan Frák, Alain Lacampagne, Giancarlo Forte, Petr Dvorak, Albano C. Meli, Vladimir Rotrekl

**Affiliations:** 1 Department of Biology, Faculty of Medicine, Masaryk University, 625 00 Brno, Czech Republic; sarka.jelinkova89@gmail.com (S.J.); petrfoj@hotmail.com (P.F.); aneta.baumeisterova@gmail.com (A.K.); aleksandra.vilotic@gmail.com (A.V.); zerzankova.lenka@gmail.com (L.M.); peslmartin@gmail.com (M.P.); t.jurakova@gmail.com (T.J.); kruta.miriama@gmail.com (M.K.); gaillyova.renata@fnbrno.cz (R.G.); Valaskova.Iveta@fnbrno.cz (I.V.); ivan.frak@gmail.com (I.F.); petr.dvorak@rect.muni.cz (P.D.); 2 International Clinical Research Center ICRC, St. Anne’s University Hospital Brno, 602 00 Brno, Czech Republic; vrbsky.jan@fnusa.cz (J.V.); giaforte@gmail.com (G.F.); 3 1st Department of Internal Medicine—Cardioangiology, Faculty of Medicine, Masaryk University, 602 00 Brno, Czech Republic; 4 Department of Clinical Genetics, University hospital Brno, 613 00 Brno, Czech Republic; 5 PhyMedExp, INSERM, University of Montpellier, CNRS, 342 95 Montpellier CEDEX 5, France; alain.lacampagne@inserm.fr

**Keywords:** DMD, dystrophin, pluripotent stem cells, genome stability, ROS, NO synthases

## Abstract

Recent data on Duchenne muscular dystrophy (DMD) show myocyte progenitor’s involvement in the disease pathology often leading to the DMD patient’s death. The molecular mechanism underlying stem cell impairment in DMD has not been described. We created dystrophin-deficient human pluripotent stem cell (hPSC) lines by reprogramming cells from two DMD patients, and also by introducing dystrophin mutation into human embryonic stem cells via CRISPR/Cas9. While dystrophin is expressed in healthy hPSC, its deficiency in DMD hPSC lines induces the release of reactive oxygen species (ROS) through dysregulated activity of all three isoforms of nitric oxide synthase (further abrev. as, NOS). NOS-induced ROS release leads to DNA damage and genomic instability in DMD hPSC. We were able to reduce both the ROS release as well as DNA damage to the level of wild-type hPSC by inhibiting NOS activity.

## 1. Introduction

Duchenne muscular dystrophy (DMD) is an X-linked rare genetic disorder which impairs muscles and results in severe disability and premature death in young men [1]. Initially, DMD starts to develop in skeletal muscles causing muscle weakness and atrophy due to fibrosis [2], preceded by the presence of apoptotic myocytes [3,4]. During teenage years, fibrosis affects also the cardiac muscle [5]. It is further exacerbated by intracardiac conduction disturbances, and it induces atrial and ventricular arrhythmias [6,7] leading to the development of dilated cardiomyopathy [8], and ultimately, to heart failure in DMD patients [5,9]. Disease progression and muscle degeneration leading to skeletal muscle wasting was recently also linked to satellite cell depletion [10,11].

DMD is caused by mutations in the dystrophin gene, resulting in truncated inactive or missing forms of dystrophin [12], which is a part of dystrophin glycoprotein complex (DGC) [13]. DGC in myocytes binds the sarcolemma to the extracellular matrix [14] and stabilizes the plasma membrane of striated muscle cells [15]. Even though a specific dystrophin isoform (Dp412) [16] and several others [17] were previously shown to be expressed in stem cells, the role of dystrophin or DGC is still unknown. DGC consists of multiple proteins, including nitric oxide synthase (NOS) isoforms [18], [19,20,21]. NOS produces NO by conversion of l-arginine to l-citruline. In low l-arginine concentration, NOS produces O_2_ or H_2_O_2_ e.g., [22,23] that acts as reactive oxygen species (ROS), or can react with NO and form ONOO, a reactive nitrogen species (RNS) that quickly decomposes to a strong oxidant HO^.^ [24]. Due to ONOO^.^ quick conversion to ROS and common strong oxidative properties, the ROS/RNS are further referenced only as ROS. The deregulation of nNOS was shown to be one of the intracellular sources of oxidative stress in the DMD animal model (*mdx*) myocytes [21]. In DMD myocytes, neuronal NOS (nNOS) loses its localization to DGC, which leads to a decrease in muscle force that can be restored by downregulation of nNOS [20]. iNOS was shown to be overexpressed in *mdx* myocytes, and this increase further regulates the expression of eNOS [25]. Increased oxidative stress in DMD myocytes was also attributed to mitochondrial complex I insufficiency [26], or changed expression of NADPH oxidase 2 (NOX2) [27]. Oxidative stress in turn leads to further interleukin-6 mediated ROS release [28,29], that initiates a vicious ROS cycle in DMD myocytes, leading to cell death and eventual myocyte replacement by scar tissue [3,4]. These pathological features resemble clinically-observed skeletal muscle and myocardial fibrosis (e.g., [30]), suggesting that myocyte depletion is associated with DMD.

Different mechanisms are proposed and still discussed to explain the discrepancy between functional impairment with premature death of DMD cardiomyocytes (CMs) [31] and the later onset of myocardial fibrosis and heart failure compared to skeletal muscle, usually diagnosed in the second decade of the patients’ lives [13]. Tissue remodeling was attributed to inflammatory response induced by the cardiomyocyte death, mediated mostly by T and B lymphocytes [32,33] and increased oxidative stress [34].

The skeletal muscle pathophysiological changes in DMD mouse models have been recently connected to satellite cell depletion [35]. The proliferation, resistance to oxidative stress, and multilineage differentiation capacities decreased rapidly in a period of weeks in mdx mice satellite cells [35], thus pointing at progenitors’ premature depletion either by cell death, differentiation or loss of self-renewal [36,37,38,39]. Insufficient data are available so far concerning the expression and function of dystrophin in stem cells [40,41,42,43], mostly due to the limited accessibility of tissue specific stem cells from patients, while animal models only partially resemble the human DMD phenotype [44]. Thus, we considered that it was of utmost importance to dissect the molecular mechanisms using a human pluripotent stem cell (hPSC) model of DMD.

## 2. Materials and Methods

### 2.1. Control Cell Lines and Cultivation

As control lines for standard model of pluripotent stem cells, we employed hESC CCTL12 (hPSCreg name MUNIe005-A, passages 39–61)) and CCTL14 (hPSCreg name MUNIe007-A, passages 23–63) derived in Masaryk University, Brno, and characterized previously [45]. hiPSC control lines are used in key experiments as additional control to limit the effect of the genetic variability of the source material. Used lines are AM13 (previously described in [46], passages 22–78), clone (cl.)1 (passages 40–89) and cl.4 (passages 62–84) obtained from Dr. Majlinda Lako (Newcastle University, UK) [47] and episomaly reprogrammed hiPSC (CBIA1, passages 44–50)) obtained from Irena Koutná (Centre for Biomedical Image Analysis, Masaryk University, Brno, Czech Republic) (described in [48]). All human pluripotent stem cell lines were routinely maintained on feeder layer of mitotically inactivated mouse embryonic fibroblasts (mEF) as described previously [46,49]. For CM differentiation, an embryoid body protocol was used as described in [50] with small modifications. For comparison of fibroblasts before reprogramming (for DMD passages 8–11), human foreskin fibroblast lines from newborns, SCRC-1041 and SCRC-1043 (passages 11–15) [obtained from the American Type Culture Collection (Manassas, VA, USA), described in [51] were used.

### 2.2. Tissue Processing, Reprogramming and Identification

The fibroblasts of two DMD patients were derived from skin/muscle biopsies with the patients informed consent and St. Anne’ University Hospital (Brno, Czech Republic) Ethics Committee approval, as previously described [52]. Briefly, the biopsy tissue was cut into 0.5–1 mm^3^ pieces and seeded onto 6 well plates in medium containing KnockOut DMEM (Invitrogen, Carlsbad, CA, USA), 10% heat-inactivated fetal bovine serum, 0.1 mM β-mercaptoethanol, 1% penicillin-streptomycin, 1% l-glutamine, 1% non-essential amino acids and layered with cover glasses. The dishes were left in the incubator for 5 days with no movement. The medium was then changed every 2–3 days and passaged first at day 10 of cultivation using trypsin.

Two DMD patient-specific human induced pluripotent stem cell lines were obtained by the reprogramming of cultivated human fibroblasts using a CytoTuneTM iPS reprogramming kit (A13780-01; Life Technologies, Carlsbad, CA, USA), according to manufacturer’s recommendations. DMD hiPSC lines are referred to as DMD02 (hPSCreg name MUNIi001-A, passages 24–85) and DMD03 (hPSCreg name MUNIi003-A, passages 17–80). DMD hiPSCs were characterized using immunocytochemical staining of pluripotency markers (Nanog, Oct4, SSEA4, TRA-1-81, for antibodies details, see Supplementary Table S10) and compared with WT hESC and WT hiPSC line. Derived mutation-carrying lines of DMD hiPSCs were verified for the mutation presence by clinical multiplex ligation-dependent probe amplification (MLPA) analysis. Analysis was performed using SALSA MLPA P034 DMD mix 1 probemix and SALSA MLPA P035 DMD mix 2 probemix (LOT B1-1014, B1-0216, MRC Holland, Amsterdam, The Netherlands), according to manufacturer’s instructions. ABI PRISM 3130 Genetic analyzer (Applied Biosystems, Foster City, CA, USA) was used to perform the experiment and data were analyzed using Coffalyser (MRC Holland, version v.140721.1958, Amsterdam, The Netherlands) software.

Short Tandem Repetition analysis for cell culture identity was performed using ABI PRISM 3130 Genetic analyzer and PowerPlex^®^ ESI17 Fast System (Promega, Madison, WI, USA) according to manufacturer’s recommendations. Karyotypes were analyzed after colchicine (2 µg/mL) incubation for 4 h, hypotonic solution treatment consisting of cultivation medium and water in ratio 1:3, bands were labeled using Giemsa staining. FISH analysis was performed using Cambio WCP 8 (SO); WCP 12 (SG) DNA Probe (Kreatech, Leica, Wetzlar, Germany). For off-target analysis, WGS libraries were prepared using NEBNext^®^ Ultra^TM^ II FS DNA Library Prep kit for Illumina (New Eengland Biolabs, Ipswich, MA, USA). Samples were sequenced at low coverage using Illumina NextSeq 500 (pair-end 2× 250 bp, with median coverage 2×). Sequences were aligned to reference genome using bwa [53] and CNVs were analyzed with Control-FREEC [54].

### 2.3. CRISPR/Cas9 DMD hESC Clone Generation

Dystrophin-deficient hESC lines were produced by CRISPR/Cas9 technology from WT hESC line CCTL14. Guiding RNA was designed to hit exon 51 of dystrophin gene. Set of complementary single-stranded DNA oligonucleotides—gRNA (DMD1_R1: 5′-CACCGCTTGGACAGAACTTACCGAC-3′, DMD1_R2: 5′-AAACGTCGGTAAGTTCTGTCCAAGC-3′) were cloned into pSpCas9(BB)-2A-GFP (PX458) (pSpCas9(BB)-2A-GFP (PX458), a gift from Feng Zhang (Addgene, Cambridge, MA, USA, plasmid # 48138) [55] and transfected into hESC line CCTL14 using FuGENE HD transfection reagent (Promega Corporation, Madison, WI, USA) according to manufacturer’s instructions. The next day, green fluorescent protein (GFP)-positive cells were FACS-sorted (MoFlo Astrios, Beckman Coulter, Brea, CA, USA) as single cells into 96-well plate and clonally propagated. 54 clones were recovered and screened for the mutation in dystrophin gene by reverse transcription PCR on mRNA level. Appropriately selected clones without signal for dystrophin were screened using MLPA analysis, which resulted in obtaining CRISPR DMD (cDMD) hESC line (for analysis used in passages 114–159).

### 2.4. Fluorescent Labeling

For detection of reactive oxygen and reactive nitrogen species (ROS/RNS), the DMD hPSC and WT hPSC cells were incubated with CellROX Green reagent (Life Technologies) for 1 h. Cells were then fixed with 4% paraformaldehyde (PFA) for 15 min at room temperature. Microscopic data were analyzed as signal intensity per area using FIJI/ImageJ [56]. All other fluorescent labeling was conducted with cells fixed by 4% PFA for 15 min in room temperature, then permeabilized with 0.5% Triton X-100 and blocked with 1% bovine serum albumin. Immunocytological analyses for pluripotent markers (Nanog, Oct4, SSEA4, TRA1-81) and cardiac markers (NOS, dystrophin, PCNA and laminB) were performed (for a complete list of antibodies and dilutions, see Supplementary Table S10). For dystrophin detection, the cells were fixed with 2% PFA and ice-cold methanol, and then incubated with preextraction buffer (25 mM HEPES, 50 mM NaCl, 1 mM EDTA, 3 mM MgCl_2_, 200 mM sucrose, 0.5% Triton X-100) prior to standard protocol for immunodetection by fluorescent labels. For nuclear counterstain, 4′,6-diamidine-2′-phenylindole dihydrochloride (DAPI, Sigma Aldrich, St. Louis, MO, USA) was used. The DNA damage was analyzed using γH2AX detection with incubation in pre-extraction buffer (25 mM HEPES, 50 mM NaCl, 1 mM EDTA, 3 mM MgCl_2_, 200 mM sucrose, 0.5% Triton X-100) for 10 min on ice prior to permeabilization. For analysis of DSB, the total number of foci per image was divided by the nuclei count per image. For double labeling of γH2AX and nNOS/iNOS/eNOS, the cells were fixed with 2% PFA and ice-cold methanol and then incubated with pre-extraction buffer. All fluorescent and confocal microscopy was performed using LSM700 confocal microscope (Carl Zeiss, Oberkochen, Germany).

### 2.5. Inhibition Conditions

To assess the influence of ROS on the formation of γH2AX foci, cells were treated for 24 h + 2 h with the ROS scavenger *N*-Acetyl-l-cysteine (NAC) at 5 mM concentration, the ˝+” indicating a medium change containing fresh NAC. To assess the effect of NOS on ROS release, cells were treated for 3 days with the NOS inhibitor Nω-nitro-l-arginine methyl ester hydrochloride (l-NAME) at a 0.1 µM concentration (N5751, Sigma Aldrich), prior to ROS/γH2AX foci analysis. For specific inhibition, l-canavanine (CAN, 100 µM, Sigma Aldrich, for iNOS), N(5)-(1-Iminoethyl)-l-ornithine HCl (l-NIO, 100 µM, Santa Cruz, for eNOS), spermine and spermidine (SM and SMD, 0,5 µM, both Sigma Aldrich, for nNOS) were used 3 days prior to γH2AX foci analysis. Silencing of each NOS isoform was performed using X-tremeGENE™ siRNA Transfection Reagent (Sigma Aldrich) and siRNA against NOS1 (nNOS, sc-29416, Santa Cruz), NOS2 (iNOS, sc-29417, Santa Cruz) or NOS3 (eNOS, sc-36093, Santa Cruz) in 250 nM concentration 48 h prior to γH2AX foci analysis. DNA damage analysis evaluation using γH2AX foci was then performed only on such colonies that showed an expression decrease compared to the non-silenced control.

### 2.6. Mutation Frequency

Mutation frequency (MF) experiments were performed on feeder-free cultures described in [46,57]. Hypoxanthine phosphoribosyltransferase (HPRT) based assays were performed to evaluate MF as described in [46] with minor modifications. Cells were seeded into media containing 2.5 µg/mL 6-thioguanine (TG; Sigma-Aldrich). The concentration of TG was increased to 8µg/mL 8 days after seeding. Mutants were selected after 3–4 weeks. MF was calculated as ratio of the number of selected mutant colonies to plating efficiency of cells in nonselective media. For induced MF, cells were irradiated by ionizing radiation (IR: 0.5 Gy/min; 137Cs; total of 3 Gy) 24 h after the initial plating. The medium was replaced every 1–2 days during a selection period of 3–4 weeks until clearly bordered colonies were manually counted. The final MFs were calculated as the ratio of the number of mutant colonies to the plating efficiency of cells in the nonselective medium at the beginning of the experiment. For MF analysis in human fibroblasts, the cells were seeded in a medium consisting of KockOut DMEM with 10% FBS, 1% l-glutamine, 1% Penicilin/streptomycin and 7 µg/mL TG and replaced every 3–4 days. Mutants were selected after 5–7 weeks.

### 2.7. Western Blot

For dystrophin immunoblots, denaturated protein samples from cell lysates were run in 4–10% gradient polyacrylamide gel for 70 min/170 V/400 mA, blotted at 100 V/300 mA for 2 h and labeled with antibody against N terminal (NCL-DYSB, Leica) and C terminal (ab15277, abcam) of dystrophin protein. As a loading control, tubulin or laminB were used. Human heart atrial tissue was obtained as procedural surplus material, available during bicaval orthotopic heart transplantation from donor organs. For western blot analysis, the sample was homogenized and dissolved in RIPA buffer (10 mM Tris-Cl (pH 8.0), 1 mM EDTA, 0.5 mM EGTA, 1% Triton X-100, 0.1% sodium deoxycholate, 0.1% SDS, 140 mM NaCl, 1 mM phenylmethanesulfonyl fluoride) and the supernatant was used.

For DNA repair protein expression, 10% polyacrylamide gel was run for 60 min/170 V/400 mA and blotted for 1 h. Proteins were labeled with antibodies against ligase1, ligase 3, ligase 4, NBS1, Rad51 and APE1 in dilution as stated in Supplementary material (Table S10).

### 2.8. Reverse Transcription PCR

Total mRNA from cell samples, human heart samples, hPSC derived hepatocytes (obtained from Tereza Váňová Ph.D) and RPMI myeloma cells (obtained from Sabina Ševčíková) was isolated using RNA Blue reagent (Top-Bio, Prague, Czech republic) according to the manufacturer’s instructions, and the total mRNA was isolated using the RNeasy Micro Kit (Qiagen, Hilden, Germany). mRNA concentration and purity was determined using NanoDrop (NanoDrop technologies, Wilmington, Germany). For reverse transcription PCR (rtPCR), cDNA was synthesized by Moloney Mouse Leukemia Virus (M-MLV) reverse transcriptase (Invitrogen, Carlsbad, CA, USA) at 37 °C for 1 h followed by 5 min at 85 °C. The consequent PCR using Taq polymerase (Top-Bio, T032) included denaturation at 94 °C for 10 min followed by cycles of 94 °C for 30 s, annealing step and extension at 72 °C for 1 min; the final extension step proceeded at 72 °C for 10 min. The PCR primers (Generi-Biotech, Hradec Kralove, Czech Republic) and annealing conditions are shown in Supplementary Table S11. The PCR product was then run in 1% agarose gel for 45 min/130 V/500 mA and photos were obtained using UV lamp DNR MiniBis Pro (Bio-Imaging Systems, Neve Yamin, Israel).

### 2.9. Statistical Analysis

Statistical evaluation was carried out in GraphPad Prism 6 software (GraphPad Software, Inc., La Jolla, CA, USA). At least 3 biological repetitions were used in each experiment, exact value for each is represented by • in each graph. Then n value, mean and standard deviation are stated in Supplementary Tables. Kolmogorov-Smirnov normality test was performed for obtained data. Student’s *t* test, one-way ANOVA with appropriate multiple comparison post hoc test or two-way ANOVA with a Tukey’s post hoc test was applied as appropriate. *p* < 0.05 was considered statistically significant. Individual test use is specified in figure legends. All measured data underwent post-hoc statistical power analysis [58] with appropriate α value (according to *p*-value determination in each experiment set) with all the power values result above 74%.

## 3. Results

### 3.1. Model DMD hPSC Cell Lines Harboring Dystrophin Mutation Are Pluripotent

**Figure 1:**
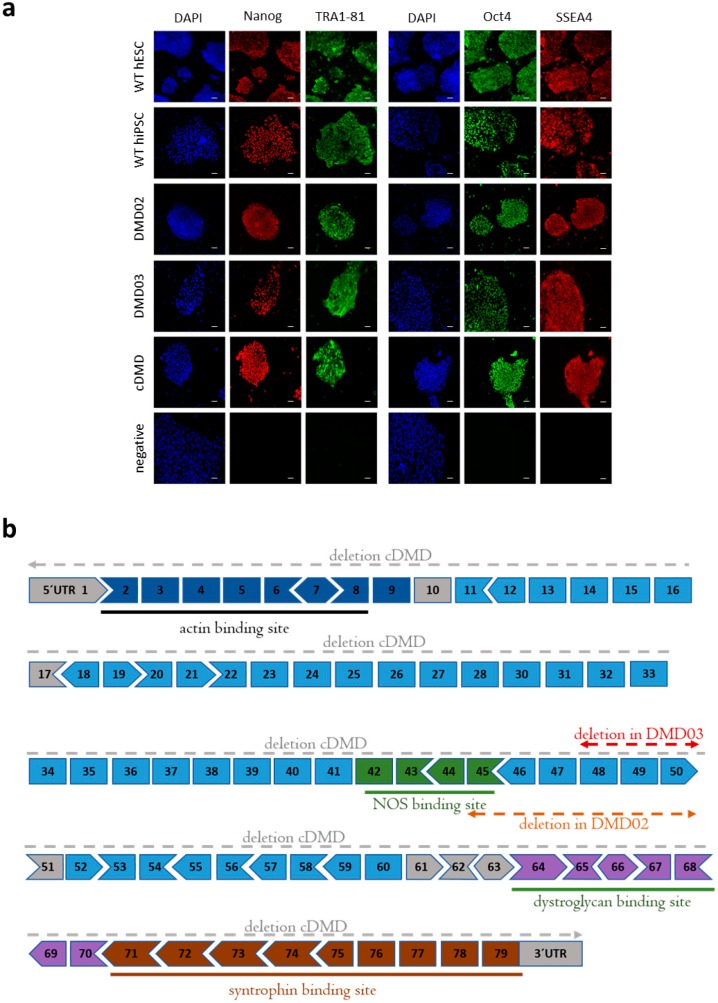
DMD hPSC lines are pluripotent. (**a**) Two patients derived (DMD02 and DMD03) and hESC derived (cDMD) DMD stem cell lines were analyzed for presence of pluripotency markers (Nanog (red on the left), TRA1-81 (green on the left) with their corresponding DAPI staining, and Oct4 (green on the right), SSEA4 (red on the right) with their corresponding DAPI staining). All DMD hiPSC and cDMD lines present comparable expression to WT (WT hESC and WT hiPSC). Nuclei are counterstained with DAPI (blue). Ruler represents 50 µm. (**b**) Structure of the DMD gene and positions of mutations in each DMD hPSC line. Locations of binding sites for actin, NOS, dystroglycans and syntrophins are shown. The diagram shows how the 79 exons fit together in terms of the normal open reading frame. Exons are colored according to the domain they encode: N-terminus (dark blue), rod domain (light blue dark green with hinge regions in grey), cysteine-rich domain (purple) and C-terminus (brown). NOS binding site is visualized in green. DMD02 line carries deletion of exons 45–50 (red line), DMD03 deletion of exons 48–50 (orange line) and cDMD lacks a whole allele and all exons are deleted from the chromosome (grey line).

Two independent DMD human induced pluripotent stem cell (hiPSC) lines DMD02 and DMD03 were derived from two patients harboring a mutation in the dystrophin gene, and individual clones were chosen empirically based on morphology (for patient data, see Supplementary Material). Both clones tested positively for pluripotent markers Nanog, Oct3/4, TRA1-81 and SSEA4 (Figure 1a) and the expression pattern was comparable to wild type (WT) hiPSC and WT human embryonic stem cell (hESC). For a complete cell line description and identification (karyotype Figure S1, STR matching Tables S1 and S2), see Cell line description and identification (Supplementary material).

The ability of DMD hiPSC to spontaneously differentiate in vitro into embryoid bodies (EBs) containing all three germ layers 15 days post aggregation was shown using reverse transcription PCR (Figure S3) and compared to WT hESC, serving as a standard of pluripotency. A similar pattern of expression change of early differentiation markers ectoderm (NEUROD1, PAX6), and mesoderm (GATA6, VIM) were found in EBs from both DMD hiPSC and WT hESC lines. Endodermal markers (AFP and GATA4) were also present in both DMD hiPSC and WT hESC lines, but AFP expression level was higher in DMD hiPSC.

The hESC line with a complete loss of one allele of DMD gene (cDMD) was obtained by CRISPR/Cas9 technology (MLPA analysis, Figures S4 and S5). The cDMD line was tested for pluripotency marker expression with positive labeling for Nanog, Oct3/4, Tra1-81 and SSEA4 (Figure 1a). For further description of the line (karyotype Figure S1, sequencing Figure S2, STR matching, and MLPA Figures S4 and S5), see Supplementary material (Cell line description and identification, Figures S1 and S2).

A complete characterization of mutation localization in each DMD hPSC line is illustrated in Figure 1b.

### 3.2. WT hPSC, But Not DMD hPSC, Express High Molecular Weight Isoform of Dystrophin

**Figure 2:**
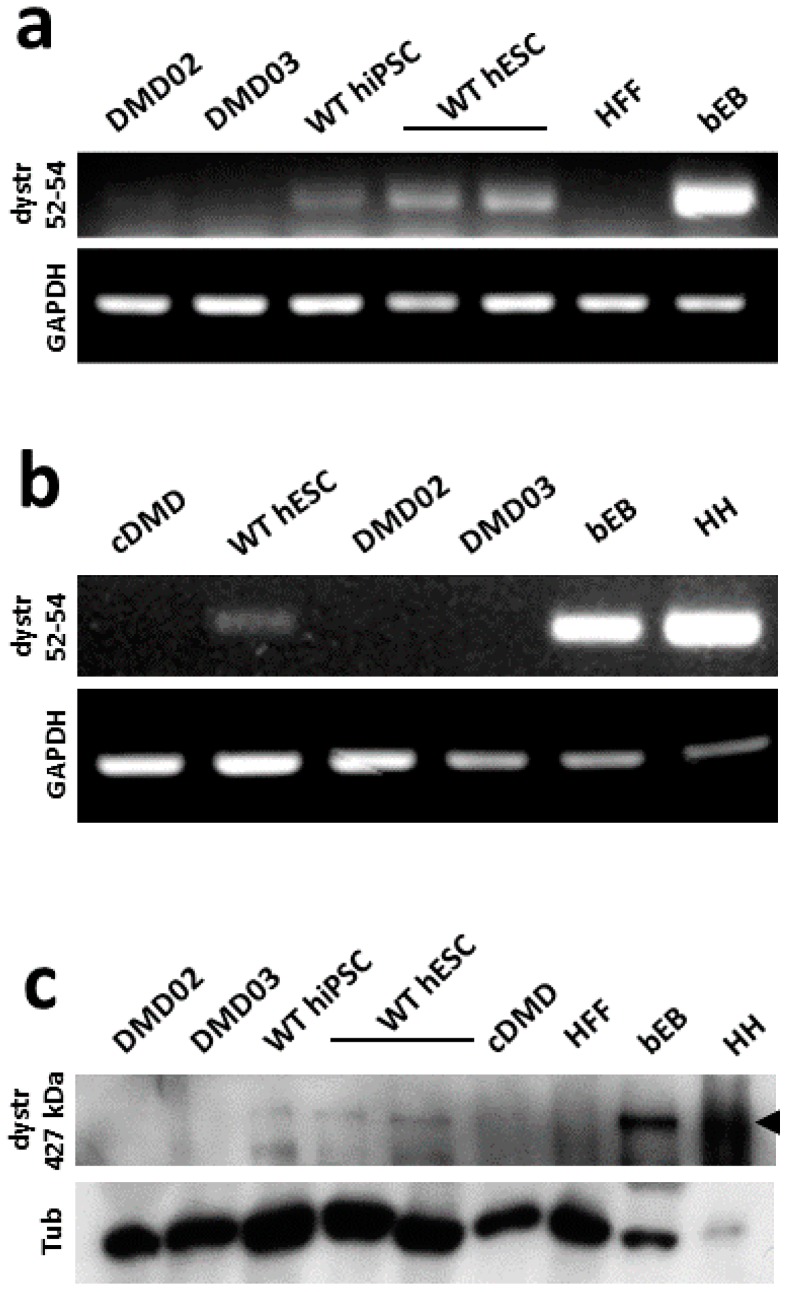
WT hPSC, but not DMD hPSC, express dystrophin. (**a**) Dystrophin mRNA exons 52–54 were detected by rtPCR in both WT hiPSC and hESC lines and in CM containing beating embryonic bodies (bEB). No signal was detected in DMD hiPSC lines. GAPDH was used as a loading control. Human foreskin fibroblasts (HFF) were used as negative control. (**b**) demonstrates the absence of expression of dystrophin mRNA exons 52–54 in cDMD line. For positive control, bEB and human heart (HH) sample were used. DMD02 and DMD03 hiPSC lines were used for lack of expression comparison. (**c**) High molecular weight dystrophin (dystr) protein (Dp427 isoform; antibody against N-terminal part) is expressed in pluripotent stem cells as shown by immunoblot of dystrophin expression in WT hiPSC and two WT hESC lines, while all DMD hPSC lines show complete lack 427 kDa form of dystrophin. As a positive control, human heart (HH) biopsy of non-dystrophic patient was used together with WT hESC derived bEB. HFF were used as a negative control. Tubulin (Tu) was used as a loading control. Loading of bEB and HH was adjusted not to overload dystrophin due to its high expression. DMD hPSC lines are pluripotent.

mRNA analysis of exons 52–54 (downstream of the patient’s mutation) revealed that all tested WT hiPSC and WT hESC expressed dystrophin mRNA (Figure 2a,b), although the expression was weaker compared to adult healthy human heart biopsy (for human heart biopsy details, see Materials) or beating cardiac EBs differentiated from hPSC.

All tested WT hPSC also expressed the high molecular weight dystrophin protein, as shown by western blot protein analysis (427 kDa; Figure 2c and Supplementary Figure S6). The DMD hiPSC derived from both patients and cDMD presented a complete loss of dystrophin expression on mRNA levels (Figure 2b) and protein levels (Figure 2c) compared to the corresponding control hPSC.

### 3.3. Dystrophin Defect Leads to Elevated Oxidative Stress in DMD hPSC

**Figure 3:**
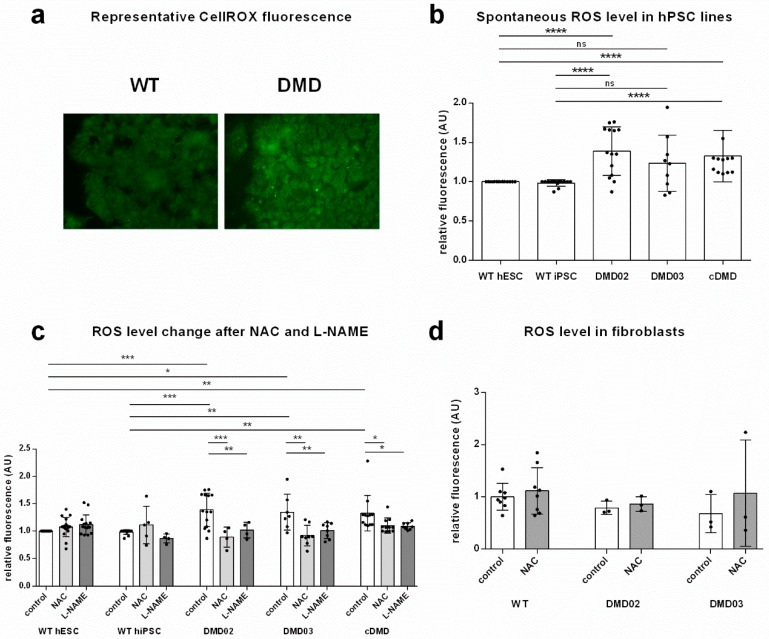
Elevated spontaneous NOS induced ROS release in DMD hPSC. (**a**) Representative example of ROS level in hiPSC line DMD03 compared to WT hESC. Fluorescence intensity per area (Image J) was normalized to WT hESC. (**b**) Spontaneous level of ROS measured by CellROX Green dye fluorescent signal in DMD hPSC lines is elevated compared to the WT hESC. Statistical significance was evaluated by One sample *t*-test comparing the values to normalized relative control in WT hESC and WT hiPSC (* *p* < 0.05, ** *p* < 0.01, *** *p* < 0.001). Error bars show standard deviation. (**c**) ROS production with ROS scavenger N-acetyl-l-cysteine (NAC) treatment was significantly decreased in all tested DMD hPSC lines but not WT hPSC lines compared to the cells with no treatment. ROS production with NOS inhibitor l-NAME decreased significantly in all DMD hPSC lines. Graph shows change in fluorescent intensity per area expressed as percentual value of untreated controls of corresponding hPSC line. Statistical difference was evaluated by two-way ANOVA comparing the NAC/l-NAME treated cells to non-treated control cells as well as WT cells compared to DMD cells. (**d**) ROS levels in DMD fibroblasts from both DMD patients used for reprogramming do not differ from three independent WT foreskin fibroblasts from newborn boys at similar passage number. Statistical difference was evaluated by one-way ANOVA. At least 3 biological repetitions were used in each experiment, exact value for each is represented by • in each graph.

Based on the observation of dystrophin expression in pluripotent stem cells and its loss due to the mutation, we asked whether dysfunctions associated with DMD muscle cells such as ROS elevation [28,34] were also present at the pluripotent state. We analyzed production of ROS in the DMD hPSC lines (DMD02, DMD03 and cDMD) using CellROX dye (Figure 3a), (detects both, ROS and RNS). All DMD hPSC lines presented significantly increased ROS production (normalized values evaluated by one sample *t* test to relative value of WT controls) (Figure 3b, Table S1). Even though a significant change in ROS presence was identified, the mRNA expression of antioxidant enzymes is not changed with the exclusion of GPX3, whose expression was elevated in all DMD lines compared to WT hPSC (Supplementary material Figure S7). Treatment with ROS scavenger N-acetyl-l-cysteine (NAC) led to a significant reduction in ROS level in DMD hiPSC lines (Figure 3c, Table S1) comparable to WT hPSC (Figure S8) while no change after NAC treatment was detected in WT hESC (Figure 3c). In order to eliminate the genetic variability of the individual cell lines from specific effect of NAC, all WT and all DMD hPSC lines were pooled together, showing that significantly elevated ROS level in DMD hPSC (*p* < 0.0001) can be reduced to the level of WT hPSC by the application of NAC (*p* = 0.6270 as evaluated by Wilcoxon test comparing control values from WT iPSC and WT hESC to corresponding NAC treated DMD lines, Figure S8, Supplementary Table S2).

In order to exclude genetic bias, DMD patient specific primary fibroblast cultures, which were used to reprogram the DMD hiPSC lines, were also tested for ROS level together with two different WT primary cell lines of human foreskin fibroblasts as controls. No differences in the ROS levels between DMD and WT fibroblasts were observed (*p* = 0.2) which corresponds to the absence of high molecular weight dystrophin expression in fibroblasts. Similarly, the NAC treatment did not have effect on ROS accumulation in WT or DMD fibroblasts (*p* = 0.7) (Figure 3d, Supplementary Table S3).

### 3.4. Inhibition of NOS Activity Led to Decrease in ROS Production in DMD hPSC But Not in WT hPSC

In order to evaluate the contribution of DGC-associated NOS on the oxidative stress in hPSC, we analyzed the ROS release in DMD hPSC lines and WT hPSC in presence and absence of Nω-nitro-l-arginine methyl ester hydrochloride (l-NAME), a non-specific NOS inhibitor (Figure 3c, Table S1). The inhibition of NOS led to significant decrease of ROS release in DMD hPSC. The resulting level of ROS in DMD hPSC with l-NAME inhibited NOS matched the level found in WT hPSC (*p* = 0.1207 as evaluated by Wilcoxon test comparing control values from WT iPSC and WT hESC to corresponding l-NAME treated DMD lines, Figure S8, Table S2). In contrast, the WT hESC showed no difference under l-NAME treatment (*p* = 0.1926), WT hiPSC showed a decrease by 10% of ROS level (Figure 3c and Figure S8).

### 3.5. NOS Activity Is Elevated in DMD hPSC Lines

**Figure 4:**
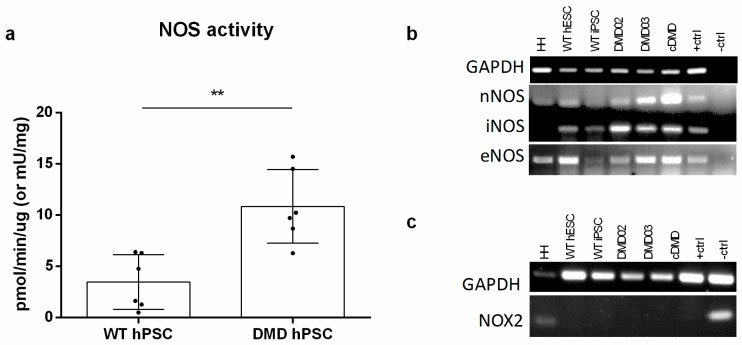
Enzymatic NOS activity is increased in DMD hPSC lines. (**a**) NOS activity assay showed increased production of NO (in pmol/min/µg) in DMD hPSC lines when compared to WT hPSC. 6 independent repetitions (for each WT and DMD hPSC) were used for the analysis. Student *t*-test was used for statistical evaluation of significance (** *p* < 0.01). Errorbars show standard deviation. (**b**) All three NOS isoforms (nNOS, iNOS and eNOS) are expressed in WT as well as in DMD hPSC. For a positive control (+ctrl), HH (for NOX and nNOS), hepatocytes (for iNOS) or RPMI myeloma cells (for eNOS) were used. Water instead of cDNA was used for negative control (−ctrl). GAPDH was used as loading control. (**c**) mRNA analysis revealed that NOX2, is not expressed in pluripotent state. Human heart tissue (HH) was used as positive control, GAPDH was used as loading control. At least 3 biological repetitions were used in each experiment, exact value for each is represented by • in each graph.

To examine if the increased ROS are caused by increased expression, or increased activity of NOS, the RNA expression and NOS activity were analyzed. The NOS activity assay showed significant increase in NOS activity in all DMD hPSC lines compared to WT hPSC lines (Figure 4a). (*p* = 0.0023). At the same time, all NOS isoforms’ (nNOS, iNOS and eNOS) mRNAs were expressed in WT and DMD hPSC (Figure 4b) without any quantitative change in expression levels (Supplementary Table S4). It ought to be said that a known ROS producer in DMD CMs, NADPH oxidase (NOX2), is not expressed in pluripotent state at all as determined by rtPCR (Figure 4c).

### 3.6. Elevated ROS Leads to Elevated DNA Damage in DMD hPSC

**Figure 5:**
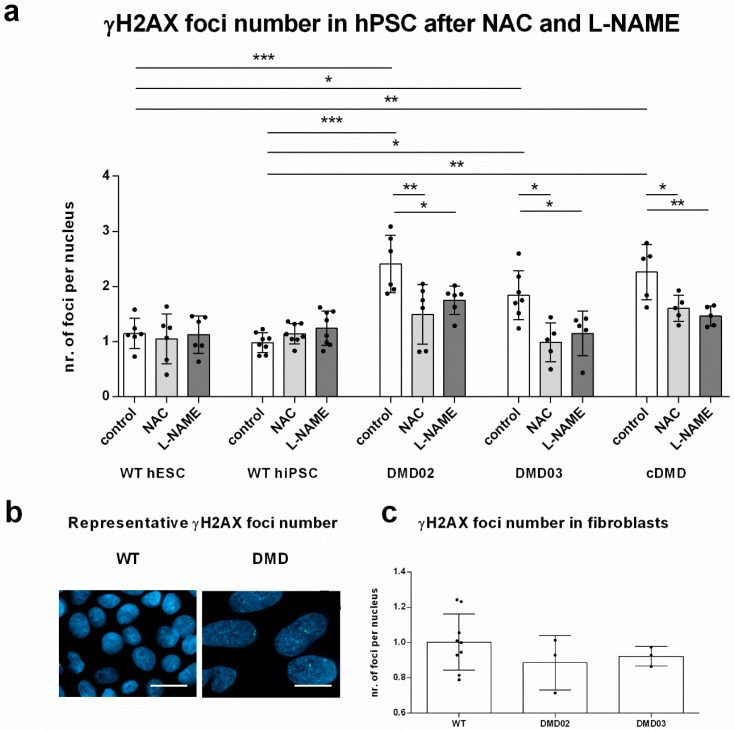
Elevated ROS level in DMD hPSC leads to DNA damage. (**a**) Numbers of γH2AX foci representing the spontaneous DNA damage in DMD02, DMD03 and cDMD hPSC lines were significantly elevated compared to WT hESC and WT hiPSC. Spontaneous formation of γH2AX foci in all DMD hPSC lines significantly decreased after the NAC treatment to the level of WT hPSC. No effect of NAC on the WT hPSC lines was observed. All DMD lines show significant decrease in γH2AX foci formation after treatment with l-NAME corresponding to the decrease detected after NAC treatment. WT hiPSC show increase after l-NAME treatment. Statistical difference was evaluated by one-way ANOVA and Sidak’s multiple comparison test (* *p* < 0.05, ** *p* < 0.01, *** *p* < 0.001) for γH2AX foci number in between different lines, and unpaired Student’s *t*-test comparing the NAC/L/NAME treated cells to corresponding untreated control cells. Error bars represent standard deviation. (**b**) Representative image of γH2AX foci staining in CCTL14 (WT) and DMD02 (DMD) hiPSC showing higher number of γH2AX foci number in DMD nuclei compared to WT nuclei. γH2AX foci are stained in green, nuclei in blue with DAPI. Line represents 20 µm. (**c**) DNA damage is not increased in DMD fibroblasts before reprogramming. γH2AX foci number per nucleus in DMD fibroblasts from both DMD patients used for reprogramming does not differ from WT foreskin fibroblasts. Statistical difference was evaluated by one-way ANOVA. The errorbars show standard deviation. At least 3 biological repetitions were used in each experiment, exact value for each is represented by • in each graph.

Our next step was to investigate the DNA damage in response to elevated ROS in DMD hPSC. We analyzed the presence of foci of histone H2A phosphorylated on serine 139 (γH2AX) associated with DNA double stranded breaks (DSB) in cell nuclei in hPSC lines (Figure 5a,b). We found a significantly elevated number of γH2AX foci per nuclei in both DMD hiPSC lines and cDMD compared to WT hPSC (for exact values, see Supplementary Table S3). Similar to ROS levels, no elevation in γH2AX foci formation was observed in the source DMD fibroblasts from the patients compared to WT fibroblasts (Figure 5c) showing that elevated γH2AX foci formation is associated with DMD hPSC cellular physiology. We also show that expression of none of the DNA repair proteins representing the major DNA repair pathways decreases specifically in DMD clones compared to WT (Supplementary Material Figure S9), suggesting that DNA repair failure does not participate on the elevated DNA damage in DMD hPSC. In order to determine the actual contribution of oxidative stress to oxidative DNA damage and the DSB formation, we analyzed the γH2AX foci in presence of ROS scavenger NAC (Figure 5a). The NAC treatment resulted in significant decrease of the γH2AX foci in DMD hPSC, while no change of γH2AX foci number was detected in WT hPSC line (Figure 5a).

### 3.7. NOS Activity Causes ROS Release Which Leads to DNA Damage in DMD hPSC

In order to establish the upstream role of NOS activity in the induction of the DNA damage in DMD hPSC, γH2AX foci formation was analyzed after l-NAME treatment (Figure 5a, exact values in Supplementary Table S3). Inhibition of NOS resulted in significant decrease in the γH2AX foci level in all tested DMD hPSC lines. Similar to NAC treatment, the WT cells did not respond to the l-NAME treatment by decrease in the γH2AX foci formation; instead, the WT hiPSC, but not WT hESC, accumulated a significantly increased number of γH2AX foci. In summary, these data show that dystrophin deficiency in hPSC leads to the production of ROS associated with NOS activity, and that this mechanism significantly contributes to the DNA DSB formation in hPSC.

### 3.8. DNA Damage Increase in DMD hPSC Is Caused by nNOS, iNOS and eNOS Isoforms of NOS

**Figure 6:**
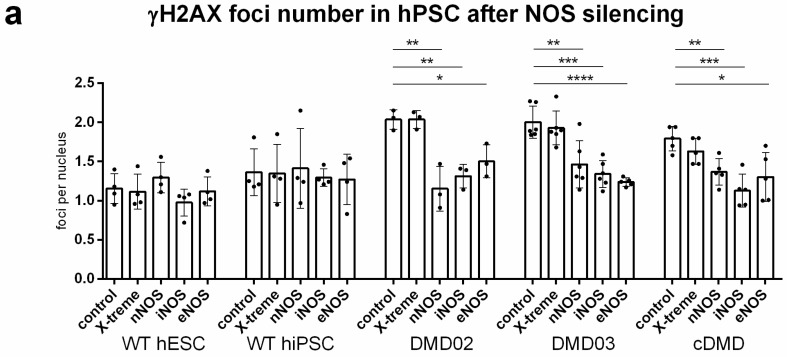

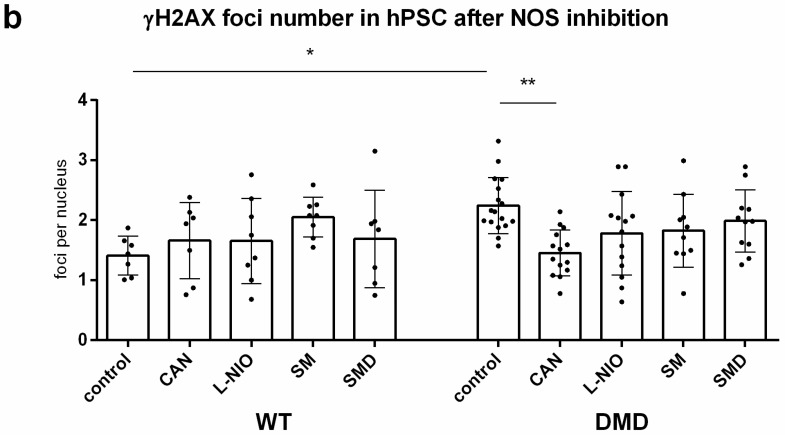
All isoforms of NOS are responsible for increased DNA damage in DMD hPSC. (**a**) DNA damage analysis after downregulation of each individual NOS isoform’s expression show significant decrease in γH2AX foci formation in DMD hPSC while no significant effect was observed in WT hPSC. Graph shows γH2AX foci per nucleus for each individual hPSC line in untreated samples (control), after application of transfection reagent (X-treme) and with application of individual siRNA (nNOS, iNOS and eNOS). Statistical difference was evaluated by two-way ANOVA and Tukey’s multiple comparison test using the mutation and treatment presence as evaluation criteria (* *p* < 0.05, ** *p* < 0.01, *** *p* < 0.001, **** *p* < 0.0001). The error bars represent standard deviation. (**b**) DNA damage analysis after application of specific inhibitors to individual NOS isoforms (CAN = l-canavanine, l-NIO = (N(5)-(1-Iminoethyl)-l-ornithine HCl), SM = spermine, SMD = spermidine) show decreasing trend in γH2AX foci presence in DMD hPSC. CAN suppressed significantly DNA damage formation in DMD hPSC cell lines while no significant effect was observed in WT hPSC. Graph shows pooled data from WT hESC and hiPSC (WT) and all 3 DMD hPSC lines (DMD). Statistical difference was evaluated by one-way ANOVA and Sidak’s multiple comparisons test; ** *p* < 0.01, the error bars represent standard deviation. At least 3 biological repetitions were used in each experiment, exact value for each is represented by • in each graph.

In order to determine which NOS isoform is responsible for ROS-induced DNA damage, all cell lines underwent treatment with siRNA against nNOS, iNOS or eNOS (Figure S10). While silencing of any of the NOS isoform in WT hPSC did not result in detectable response in γH2AX foci number, all DMD hPSC lines showed significant decrease in γH2AX foci formation after individual silencing of separate NOS isoforms (Figure 6a and Figure S11, Supplementary Tables S8–S10). All WT and DMD hPSC lines were then treated with a set of selective inhibitors prior to γH2AX foci analysis. iNOS inhibitor l-canavanine (CAN), eNOS inhibitor N(5)-(1-Iminoethyl)-l-ornithine HCl (l-NIO) and two selective inhibitors of nNOS spermine (SM) and spermidine (SMD) were used. In DMD hPSC, all inhibitors showed a decreasing trend in γH2AX foci formation with l-NIO and SM presenting a significant decrease in DMD02 and cDMD, respectively (Figure S12, Supplementary Table S7), but only CAN presented a significant change in all DMD hPSC lines (Figure 6b and Figure S12, for exact values see Supplementary Tables S9 and S10). In WT hPSC, only SM showed an increase in γH2AX foci formation in separate lines (Figure S12, Supplementary Table S7), but with no significant change overall when data from WT hPSC were pooled (Figure 6b, Supplementary Table S6).

### 3.9. Dystrophin Defect Leads to Compromised Genomic Stability of hPSC

**Figure 7:**
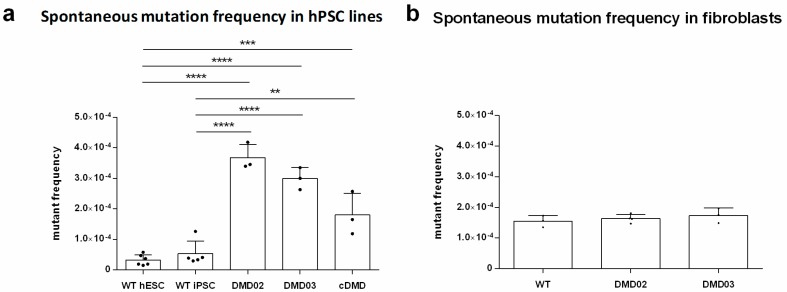
DMD hPSC lines present increased mutation frequency (MF). (**a**) MF of both patient specific hiPSC lines (DMD02 and DMD03), cDMD line and WT hESC and hiPSC line were measured using HPRT reporter assay. The graph shows significantly elevated spontaneous MF in all DMD hPSC lines compared to both WT hPSC lines. (**b**) The parental patient specific fibroblasts (DMD02 and DMD03) were compared to WT human foreskin fibroblasts of similar passage to exclude artificial effect of present MF before the reprogramming. No statistical difference was found in between all analyzed fibroblast lines. Statistical difference was evaluated by one-way ANOVA and Sidak’s multiple comparison test (* *p* < 0.05, ** *p* < 0.01, *** *p* < 0.001, **** *p* < 0.0001). The error bars represent standard deviation. At least 3 biological repetitions were used in each experiment, exact value for each is represented by • in each graph.

We analyzed the mutation frequency (MF) of all DMD hPSC lines and compared it to that of WT hPSC using our HPRT based assay, developed previously [46]. We found that the MF in DMD hPSC was significantly elevated, differing approximately one order of magnitude from both WT hPSC (Figure 7). Induction of DNA damage with ionizing radiation (3 Gy) increased MF in all tested cell lines, preserving the difference between the DMD hPSC and WT hPSC, suggesting a cumulative effect of ROS and IR induced DNA damage on the MF (Figure S13, Supplementary Table S8). No elevation in MF was observed in the source DMD fibroblasts from the patients compared to WT fibroblasts (*p* = 0.49) (Figure 7b), showing that elevated mutagenesis is specifically associated with stem cells, presumably because fibroblasts do not express high molecular mass isoforms of dystrophin as visible in immunoblots. In summary, we show that NOS activity in dystrophin deficient DMD hPSC leads to elevated ROS and DNA damage, which eventually leads to the deterioration of genomic stability in DMD hPSC.

## 4. Discussion

A few cellular models of human DMD hiPSC have been introduced recently [59,60] and reviewed in [61], and their features with regard to dystrophin deficiency have not been properly explored so far. We have obtained two independent DMD iPSC lines derived from two DMD patients which were compared to unrelated WT hiPSC and WT hESC line. We further generated cDMD hESC line by introducing DMD gene deletion in healthy hESC line using CRISPR/Cas9 strategy in order to provide a pair of isogenic cell lines, where one of them lacks dystrophin expression to eliminate the bias of varying genetic background. Our findings demonstrate for the first time aberrant phenotype of the DMD hiPSC lines and cDMD compared to WT cell lines associating the described phenotype to dystrophin expression, rather than to varying genetic background [62] or heterogeneity of different hiPSC lines [63].

Even though the molecular mechanisms causing the DMD CM death are somewhat described, no sufficient explanation on delayed myopathy development leading to muscle wasting has been offered. Recently, the skeletal muscle progenitor satellite cells [36] were also shown to express dystrophin. The absence of dystrophin expression in mice led to defects in asymmetric division [36], and skeletal muscle degeneration was shown to be a consequence of satellite cell depletion [35]. Impaired self-renewal, earlier senescence, differentiation and homing defects were also reported for cardiac progenitors in GRMD dog model [64].

For the first time, we show that hPSC expresses various forms of dystrophin, including the high molecular weight isoform, which was previously associated with myocytes [36]. These data suggest that dystrophin might play a crucial role in the stem or progenitor cell pool, long before definite differentiation into skeletal myotubes or CMs. Data on the dystrophic progenitor phenotype together with expression of dystrophin in hPSC suggest that the general stem cell population might be vulnerable to DMD-related damage, and that hPSC lacking dystrophin might serve as valid model for molecular mechanisms leading to DMD related stem cell phenotype. The depletion of *mdx* mouse satellite cells has been connected to up-regulation of Notch and pro-inflammatory signaling molecules in skeletal muscle [65]. The effect of Notch signaling on the progenitor fate decision was shown to be associated with elevated level of ROS, such as that in lamellocytes [66]. ROS were shown to induce loss of stemness [67] and cell fate change [68] via increased DNA damage and the activation of checkpoint mechanisms [67]. ROS were also shown to contribute to the pathophysiology of muscular dystrophies [3], as well as to DMD [69,70]. We extend this knowledge by showing that not only CMs [28,71,72,73], but already DMD hPSC suffer from elevated ROS levels due to increased NOS activity. ROS readily induces DNA mutations [74] via oxidative DNA damage [75,76]. The ROS-induced DNA base damage is readily converted by base excision repair to DSB in hPSC [77], triggering histone H2A phosphorylation (γH2AX) and compromising the genomic stability of hPSC [46]. Our data show elevated DNA DSB (detected by γH2AX) in DMD hPSC. The ability of antioxidant NAC to downregulate the DNA DSB in DMD hPSC indicates that DSBs originate from ROS-induced DNA damage. We further show that ROS-induced DNA damage is reflected by elevated MF in DMD hPSC, which is in agreement with the concept of oxidative DNA damage mutagenicity [78]. The NOS-induced, ROS-mediated stem cell genome destabilization might also help to explain increased susceptibility of DMD patients to malignant sarcoma [79], which was previously also associated with elevated DNA damage [80]. DNA damage was shown in hematopoietic stem cells to drive their differentiation into terminally-differentiated cells [38]. We suggest that the DNA damage and mutagenesis shown in DMD hPSC might lead to diminished stem cell potential and stem cells’ ageing (reviewed e.g., [81]), and subsequent stem cell depletion.

Mitochondrial damage [82] due to ROS production in the *mdx* murine myocytes was in the past attributed to mitochondrial ROS release [26]. In this context, all three isoforms of NOS seem to have a physiological role in myocytes, including CMs [73]. All three isoforms seem to be capable of interaction with DGC via 16–17 spectrin-like repeat of dystrophin (coded by exons 42–45,) but also via PDZ domain on α1-syntrophin [83] or through caveolin interaction [84]. The loss of dystrophin results in the increase of NOS activity, as shown by comparing the NOS activity in WT and DMD hPSC. Further inhibition of NOS was shown to block the synergistic effect of mechanical and oxidative stress on sarcolemmal damage in *mdx* mice [85], implicating NOS in oxidative stress regulation. Active endothelial NOS (eNOS) has been previously shown to be associated with attenuated superoxide production [86]. nNOS or inducible NOS (iNOS) deregulation were also shown to be associated with elevated ROS levels in myocytes [21,73,87]. iNOS has been previously shown to be overexpressed in myocytes in response to DMD pathophysiology [88]. We showed that inhibiting the general NOS activity in DMD hPSC leads to decreased ROS level, and consequently, to decreased DNA damage level. The effect was dystrophin specific and not caused by genetic variability or reprogramming, as it was neither detected in the WT hPSC nor in the donor fibroblasts. Only in WT hiPSC, the l-NAME treatment lowered ROS level and significantly increased the detected DNA damage. This might have only limited biological relevance, because the change is small (up to 30%) compared to DMD cell lines, and WT hESC did not respond to the treatment at all. Individual silencing of all NOS isoforms using specific siRNA led in all three cases to a significant decrease in γH2AX foci formation in DMD hPSC, proving that each of the NOS isoform plays a role in regulation of ROS production and consequent DNA damage.

Inhibition of individual NOS isoforms by specific inhibitors also resulted in a decrease in ROS production, as well as γH2AX foci formation specific to DMD cell lines; however, except for CAN on γH2AX foci formation, these results did not reach significance. This can be explained by the incomplete inhibition or incomplete penetration of the inhibitors to the cells, but also by their off-target effect, such as, for example, the capacity of l-NIO/SM and SMD to regulate processes such as cell proliferation and differentiation [89,90] or DNA binding [91]. It has been shown that nNOS is bound to dystrophin through its PDZ domain [83,92], and eNOS is binding to DGC through caveolin [93]. Also, higher activity of eNOS has been shown when dissociated from caveolin [94,95], and changed nNOS activity in *mdx* CMs has been reported [19]. Our work shows that a lack of dystrophin leads to the loss of dystrophin-associated regulatory function on nNOS, eNOS and iNOS activity triggering ROS release and consecutive DNA damage. ROS and DNA damage increase in DMD hPSC. But the expression of antioxidant enzymes with an exception in GPX3, that is increased in DMD hPSC, did not decrease. Also the expression of the major DNA repair proteins was unaltered in DMD hPSC. It is thus unlikely that these processes participate in the oxidative DNA damage observed in DMD hPSC. This suggests that the failure of dystrophin to organize the dystrophin-associated proteins binding NOS deregulates ROS producing activities in hPSC similarly to dystrophic CMs [21,73]. These results also explain the failure of an attempt to compensate the nNOS by nitrate supplementation, which had detrimental effect on *mdx* mice muscle, presumably by even further elevating oxidative stress [96]. It must be noted that the NOS-mediated ROS production may be just part of total ROS release, which was previously associated also with mitochondrial complex-I insufficiency [26], but it might also lead to a ROS vicious cycle mediated by NF-kB induction [28], shown in *mdx* mice skeletal muscle. Since we showed that hPSCs do not express any NOX2, a main ROS producer in myocytes and CMs in DMD [27,28], it is unlikely that NOX2 would be responsible for the ROS production in pluripotent state.

In summary, our results suggest, for the first time, that dystrophin deficiency in DMD affects the genomic stability of human DMD hPSC. We propose that the absence of dystrophin in stem cells leads to the deregulation of NOS, which leads to ROS upregulation, DNA damage and mutagenesis. That, in turn, can be reflected by genomic instability-driven differentiation of the progenitors similar to human mammary epithelial cells [97], mice hematopoietic stem cells [38,98] or hair follicle [37] stem cells. However, while it is not clear whether the DMD-associated hPSC phenotype will be mimicked by tissue stem cells/progenitor impairment, we hypothesize, that progressive DNA damage/mutagenesis-driven depletion of stem cell population would explain the development of delayed muscle impairment associated with Duchenne muscular dystrophy.

## Supplementary Materials

The following are available online. Patient data and diagnosis; Cell line description and identification; Figure S1: Karyotypes of used DMD lines and their parental source cells. Figure S2: Analysis of mutations induced by off target cleavage by DMD targeted CRISPR/Cas9 by whole genome sequencing and subsequent alignment; Figure S3: DMD hPSC do not differ in their differentiation capacity from WT hESC in in vitro spontaneous differentiation assay.; Figures S4 and S5: MLPA analysis of DMD patients derived hiPSC and cDMD; Figure S6: hPSC express various isoforms of dystrophin; Figure S7: Antioxidant enzyme expression level is not changed; Figure S8: The spontaneous level of ROS in DMD hPSC lines was significantly elevated compared to the WT hPSC; Figure S9: Protein expression of DNA repair proteins does not change in DMD hPSC; Figure S10: Expression downregulation efficiency of iNOS, nNOS and eNOS using siRNA; Figure S11: Individual downregulation if iNOS, nNOS and eNOS using siRNA leads to decreased DNA damage. Figure S12: Use of nNOS, iNOS and eNOS specific inhibitors decreases DNA damage in DMD hPSC only partially. Figure S13: Spontaneous and IR induced mutation frequency (MF) is elevated in DMD hPSC; Table S1: Relative fluorescence of ROS analyzed by CellROX labeling and image analysis; Table S2: Pooled data for WT and DMD hPSC with relative fluorescence by CellROX labeling and image analysis; Table S3: Exact values of γH2AX foci per nuclei with N-acetyl cysteine and l-NAME treatment; Table S4: Quantification of NOS isoforms expression; Table S5: Exact values of γH2AX foci per nuclei after specific NOS silencing; Table S6: Exact values of γH2AX foci per nuclei after specific NOS silencing, pooled data for WT and DMD hPSC; Table S7: Pooled data for WT and DMD hPSC with γH2AX foci per nuclei with specific NOS inhibitors; Table S8: Exact values of γH2AX foci per nuclei with specific NOS inhibitors; Table S9: Mean values of mutation frequencies in separate lines; Table S10: Antibodies; Table S11: PCR primers and cycling conditions.
