# Supplementary material for: Dystrophin Deficiency Leads to Genomic Instability in Human Pluripotent Stem Cells via NO Synthase-Induced Oxidative Stress
Source: Cells. 2019 Jan 15;8(1):53. doi: 10.3390/cells8010053 (PMC6356905; doi:10.3390/cells8010053)
Supplement: Supplementary file 1 [file cells-08-00053-s001.pdf]

Supplementary Information:

<u>Patient data and diagnosis</u>

The first patient, born in 2002, was referred for muscle biopsy and genetic sampling due to high levels of Creatine Kinase in 2003. Initial genetic analyses at age of 12 months revealed large exon deletion behind exon 44. Until this time patient reached many gross motor skill milestones, such as holding his head up, rolling over, sitting, and standing, at normal times. Motoric abnormalities were observed at the age of 2 years, involving suspected lordotic posture while standing. Gowers sign was noted at the age of 4 years. Proximal musculature of arms, pelvis and legs was progressively weakening. Additional genetic analyses were performed at age of 6, using Multiplex Ligation-dependent Probe Amplification (MLPA) and deletion of exon 45-50 (DelEX 45-50) was observed. As confirmation also mRNA test of dystrophin gene was performed in patient and mother, resulting in occurring deletion in nucleotide position 6439-7309 – corresponding to 45-50 exons, causing shortened mRNA and truncated protein. At the age of 6 years a rapid decrease in 6 minute walking test was recorded and by the age of 9, the patient was confined to wheelchair / motorized chair and remained dependent on this support. Dyspnea and ankle swelling was observed recently (age 12). Cardiac magnetic resonance was performed, yet without significant decrease in ejection fraction. Also the echocardiographic parameters were normal. Currently the patient remains in care of dedicated specialists, without need for permanent ventilation support.

The second patient born in 1998 was examined because of suspicion for delayed motoric development, suspected Gowers sign and progressing lordosis. Conducted was DNA analysis which found deletion of exons 45-49 and 50 in dystrophin gene. MLPA analysis was conducted in 2010 and confirmed previous findings. Since the age of 7 the patient is bound to wheelchair. Dyspnea was observed infrequently and recently ventilator support during the sleep period had to be applied (age 16).

Cell line description and identification

The DMD patient specific hiPSC lines are karyotypicaly healthy (both 46, XY, Supplementary Fig1) and short tandem repeats analysis (unpublished data) confirmed their origin from the patient derived fibroblasts. MLPA analysis showed presence of the same mutation in the DMD hiPSC lines as was previously detected in the patients (patient 1: deleted exons 45-50, referred to as DMD02; and patient 2: deleted exons 48-50, referred to as DMD03; see Fig. S2 and S3). The pluripotency of both DMD hiPSC lines was confirmed by in vitro spontaneous differentiation assay showing presence of early markers of ectoderm, mesoderm and endoderm (Supplementary Figure 2.)

The CRISPR mutated cDMD hESC line was derived from CCTL14 WT ESC line, 46, XX, der(18), with a derived chromosome 18. After CRISPR/Cas9 DMD targeted mutation introduction, the derived chromosome 18 remained present in the cDMD hESC (46X, del(X), der(8), t(8;12). The CRISPR/Cas9 targeting resulted in a complete deletion of p arm of one of the X chromosomes as visible in the karyotypes (Fig. S1) which resulted in a complete loss of one allele of DMD gene. This mutation

lead to a complete loss of dystrophin mRNA and protein expression (Figure 1). The karyotype analysis also revealed a reciprocal translocation of chromosomes 8 and 12 (confirmed by FISH, Fig. S1). No other changes were detected via karyotyping. Whole genome sequencing based copy number variation comparisons between cDMD and CCTL14 hESC lines confirmed deletion on one copy of chromosome X p arm. An alignment to genome (hg19) showed additional mutation on chromosome 18, present in both the source CCTL14 and cDMD. No other changes were detected with used resolution, as the reciprocal translocation found by FISH analysis is compensated.

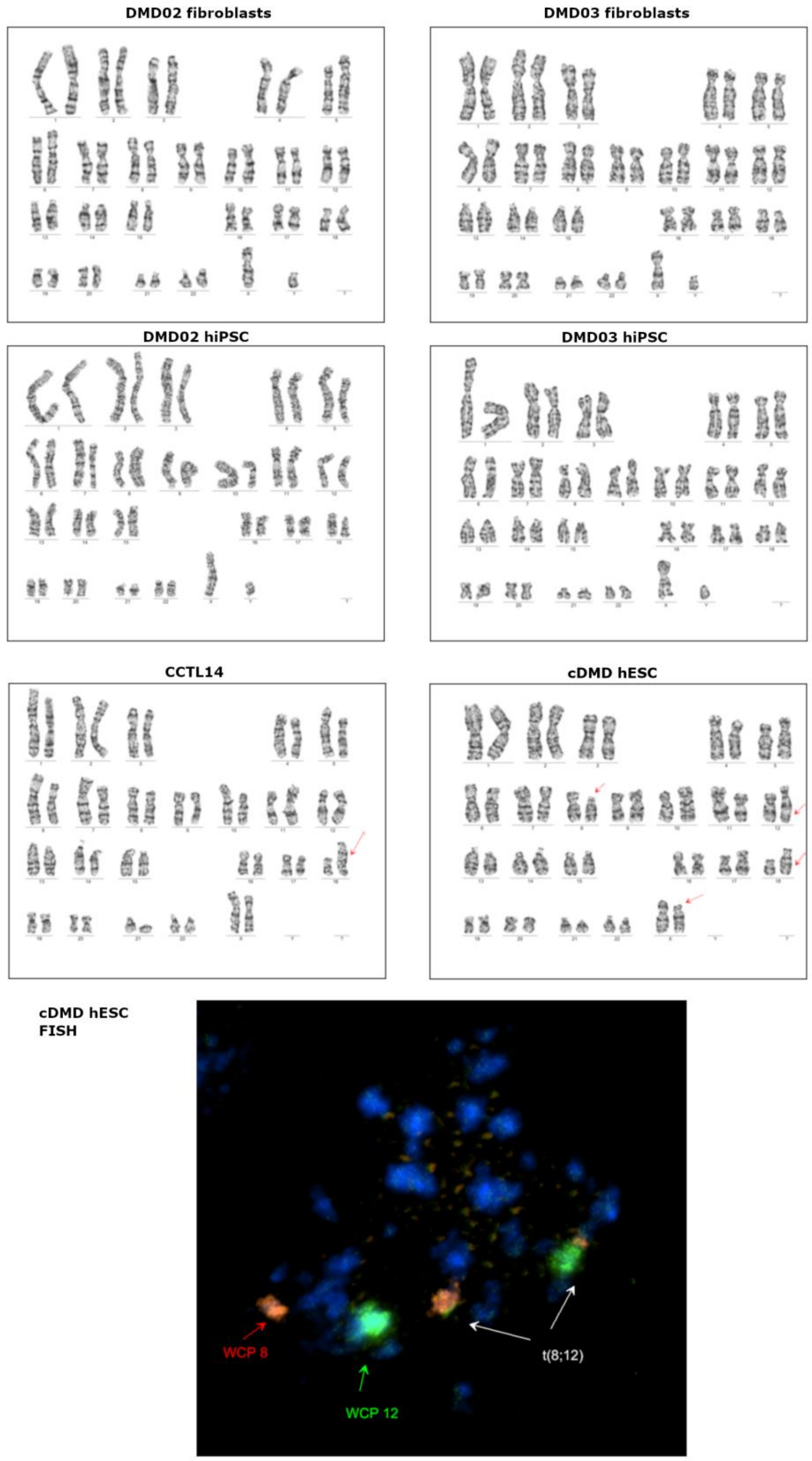

Fig. S1: Karyotypes of used DMD lines and their parental source cells. Karyotype

analysis show healthy male karyotype for both DMD02 and DMD03 hiPSC lines (46, XY) similarly to the original fibroblasts. cDMD line was derived from CCTL14 line that had karyotype 46, XX, der(18). Next to this change, the cDMD showed karyotype 46, X, del(X), der(18), t(8;12) with reciprocal translocation confirmed by FISH. The reciprocal translocation of chromosomes 8 and 12 was probably due to off target cleavage of the CRISPR gRNA used for the mutation introduction.

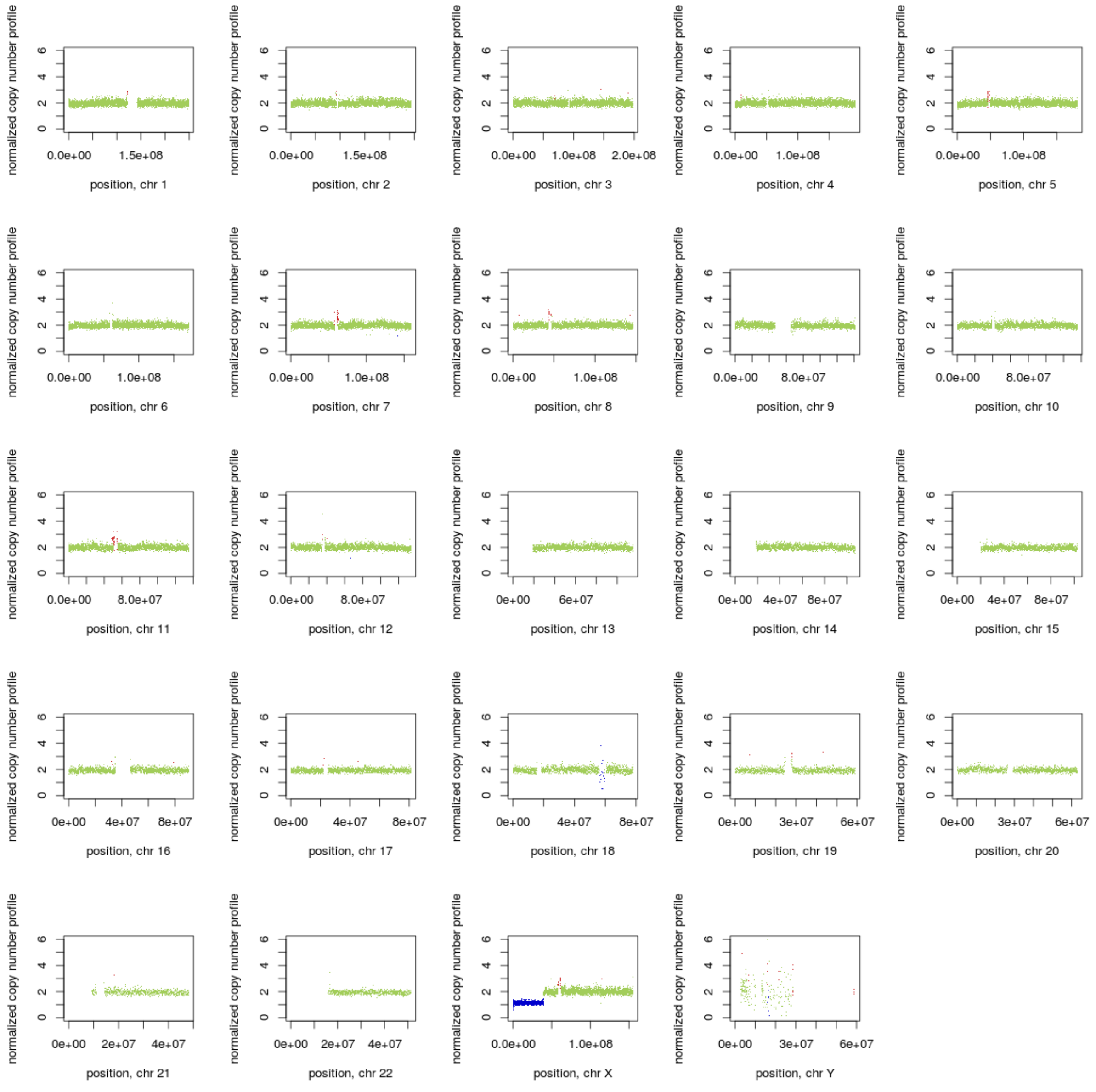

Fig. S2: Analysis of mutations induced by off target cleavage by DMD targeted CRISPR/Cas9 by whole genome sequencing and subsequent alignment. The sequencing and comparison of CCL14 and cDMD confirmed the existence of only single target presented as deletion on chromosome X beyond DMD gene locus. No other off targets were identified.

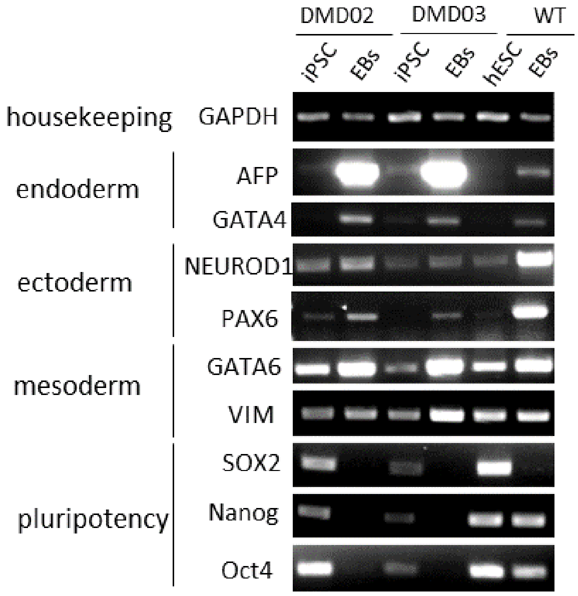

Fig. S3: DMD hPSC do not differ in their differentiation capacity from WT hESC in i*n vitro* spontaneous differentiation assay. Spontaneously formed EBs were analyzed by rtPCR for presence of cell types from all three germ layers. GAPDH was used as loading control. AFP and GATA4 were used as markers of endoderm, NEUROD1, PAX6 as markers of ectoderm, vimentin (VIM) and GATA 6 as mesodermal markers and Oct4, Nanog and SOX2 as pluripotency markers.

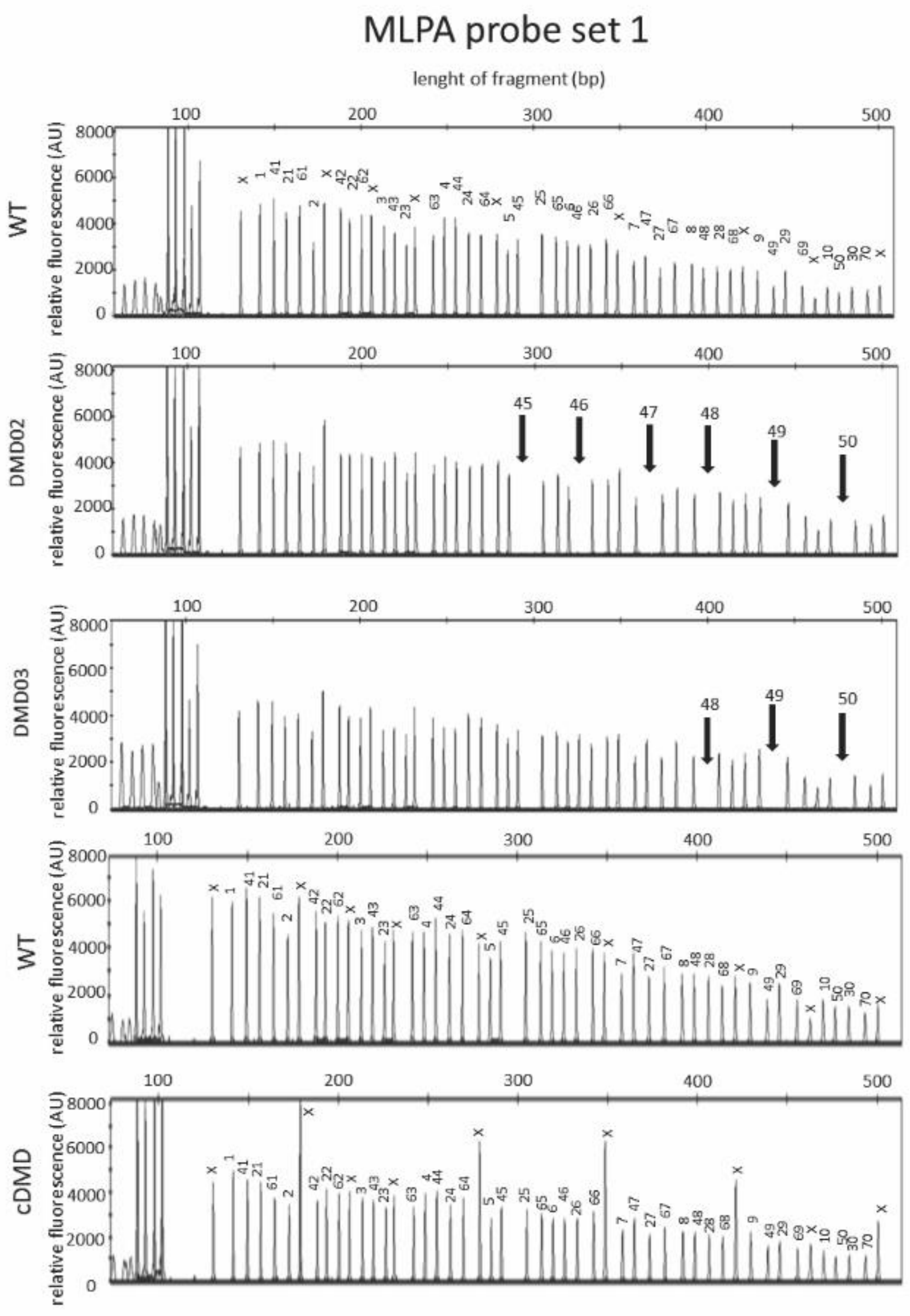
MLPA probe set 1
lenght of fragment (bp)
WT
DMD02
DMD03
WT
cDMD
relative fluorescence (AU)
45
46
47
48
49
50
48
49
50

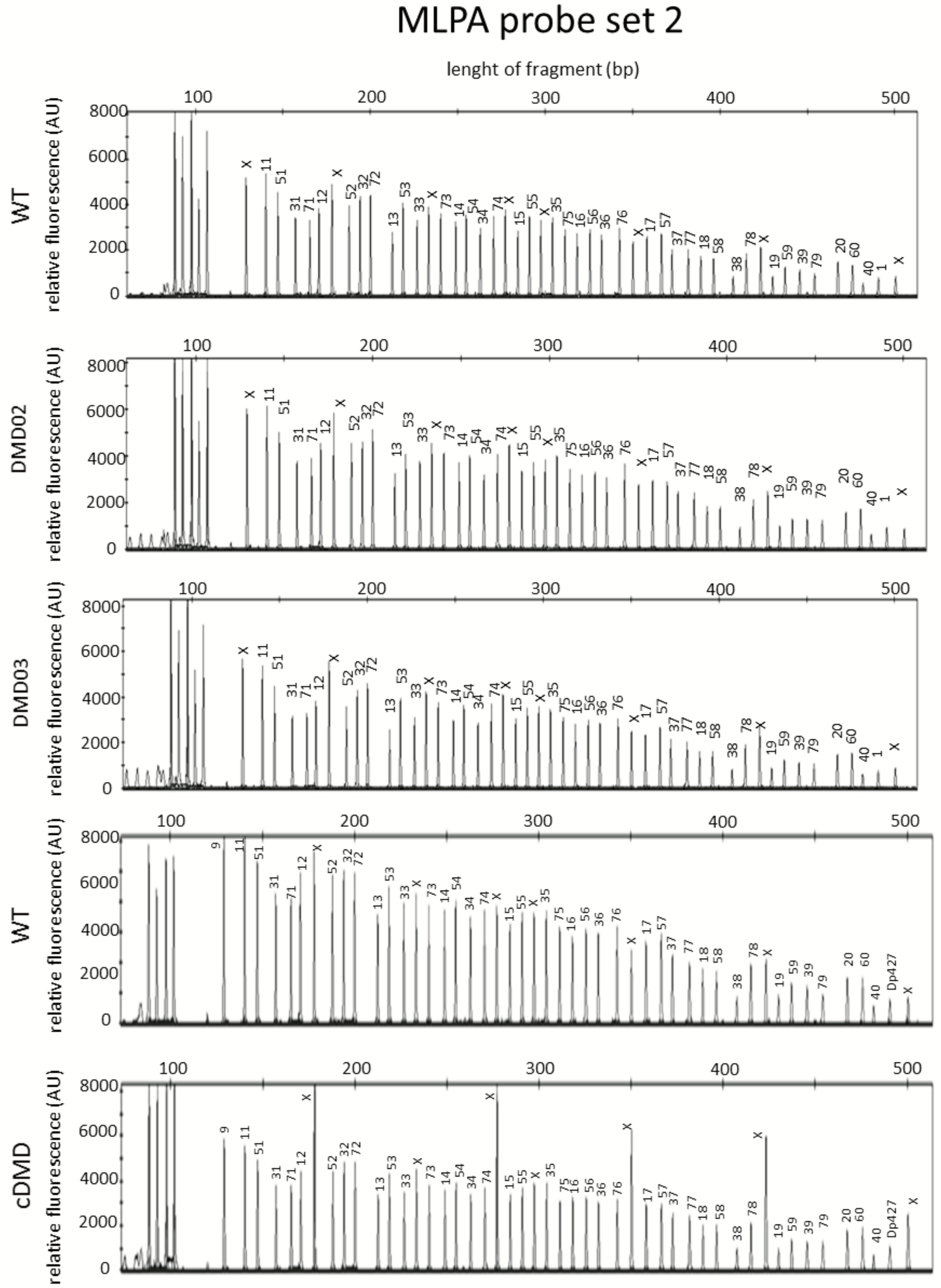

Fig. S4, S5: MLPA analysis of DMD patients derived hiPSC and cDMD. The MLPA analysis revealed deletion of exons 45-50 and exons 48-50 in DMD02 and DMD03 hiPSC lines, respectively, and a complete loss of one allele in cDMD. The WT panel shows exon peaks annotation for easier orientation and identification of deleted exons.

Missing peak stands for missing exon (arrows), lower peak (cDMD: all peaks) stands for deletion in one of the two copies of the gene. (S2) shows first probe set for DMD gene, (S3) shows second set of probes.

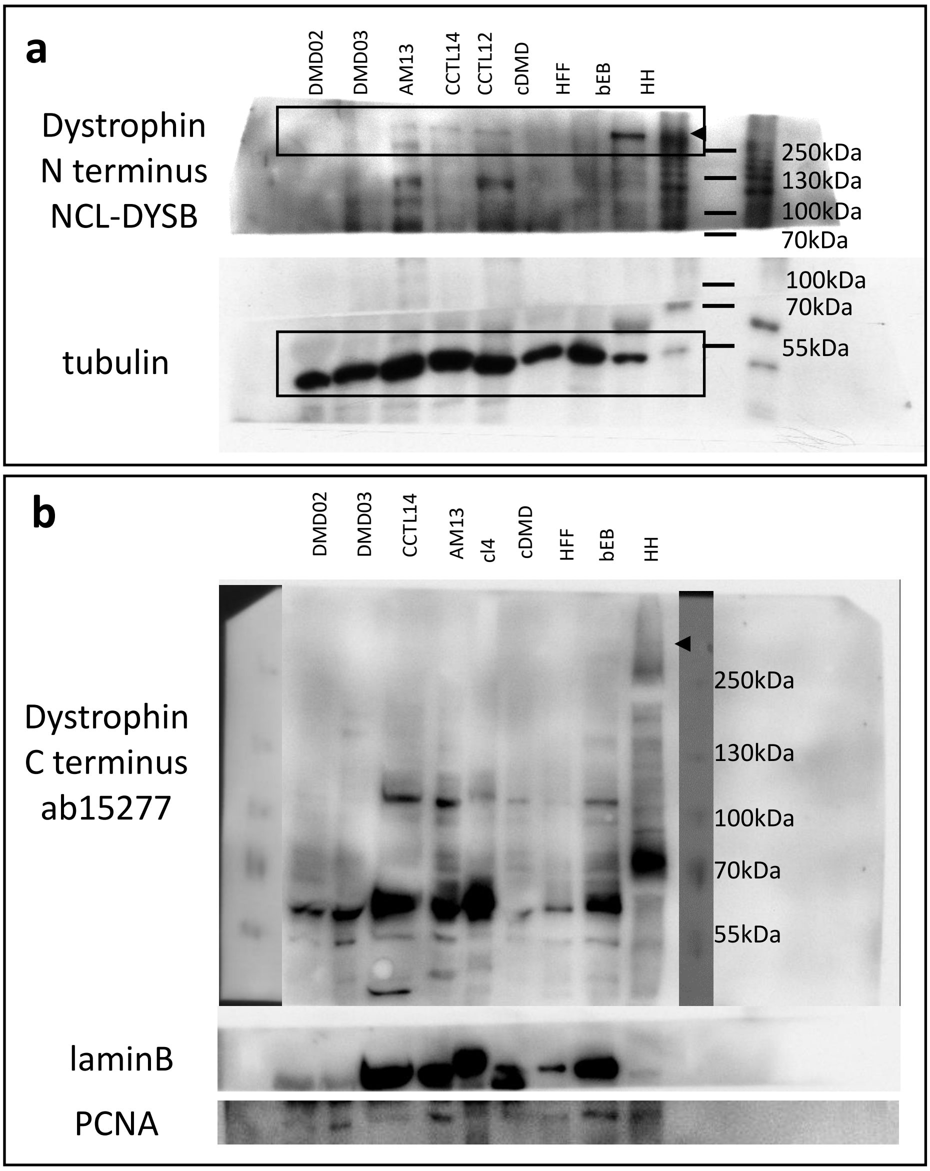

Fig. S6: <u>hPSC express various isoforms of dystrophin.</u> WT hPSC express various isoforms of dystrophin as represented by use of antibody recognizing N-terminus of dystrophin protein (NCL-DYSB, Leica, aa residue 321-494) (a), and antibody recognizing C-terminus of dystrophin protein (ab15277, abcam, aa residue 3650) (b). (a) Several isoforms of dystrophin were recognized above 70kDa by N-terminus antibody including the Dp427 (arrow) high molecular weight (framed; this section also represented in the main text of this manuscript). For loading control, the membrane

was cut above 70kDa mark and labeled with tubulin antibody. (b) The C terminus antibody does not recognize well the long isoforms of antibody with a very faint signal at area of 427kDa (arrow). The pattern of the isoform expression varies strongly in the samples of human heart tissue, CM containing spontaneuosly contracting (or beating) embryoid bodies (bEB) and the hPSC samples. Membrane was first whole labeled with dystrophin antibody, then stripped and labeled again, this time with laminB antibody and PCNA as loading control and nonskeletal protein.

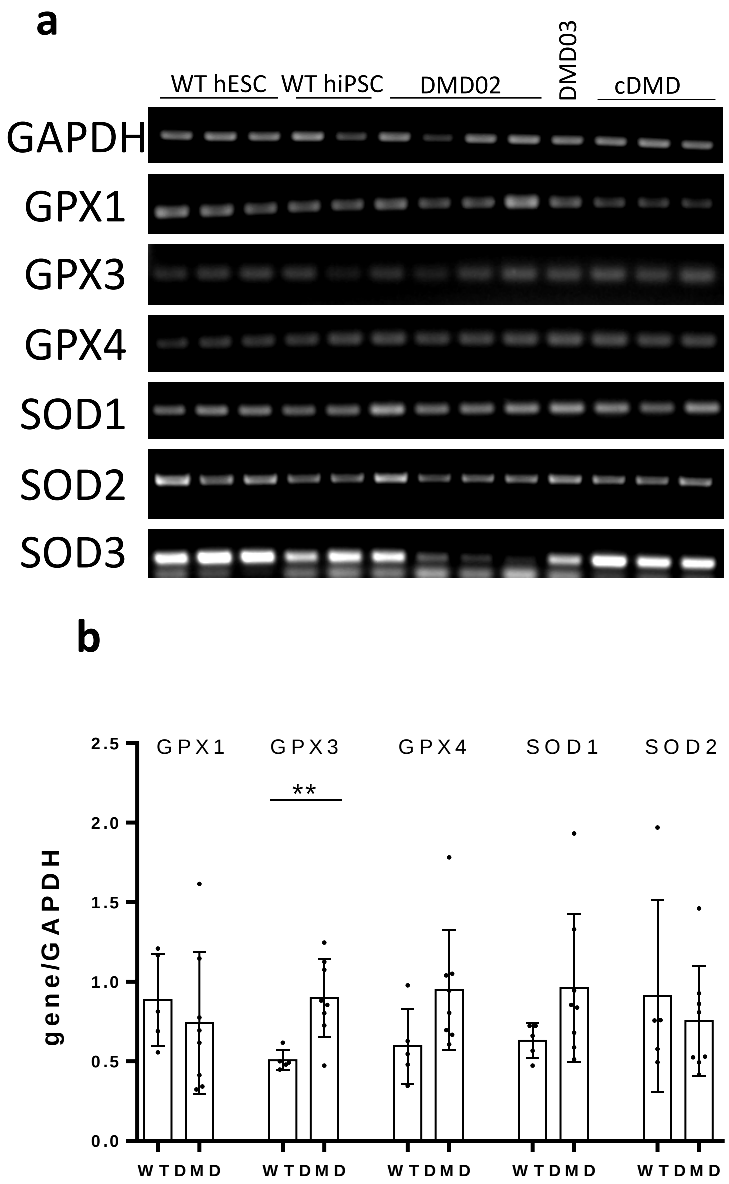

Fig. S7: Antioxidant enzyme expression level is not changed. (a) Semi-quantitative reverse transcription PCR of various line samples show that antioxidant enzymes expression does not change with the exception of Glutathione peroxidase 1 (GPX3). Glutathione peroxidase 1 and 4 (GPX 1, GPX4), superoxide dismutase 1, 2 and 3 (SOD1, SOD2, SOD3 show no significant change in expression between WT and DMD hPSC lines. SOD3 seems to be downregulated in DMD02 samples, but the change is strictly

line specific, not mutation presence specific. GPX3 mRNA expression is marginally increased in DMD hPSC. Each band represents individual independent biological repetition. GAPDH serves as loading control. (b) Densitometry of each preparation compared to GAPDH expression shows quantitative differences between WT and DMD hPSC lines in expression of each observed antioxidative enzyme. Errorbars represent standard deviation, statistical difference was calculate using Student t-test (**p<0,01).

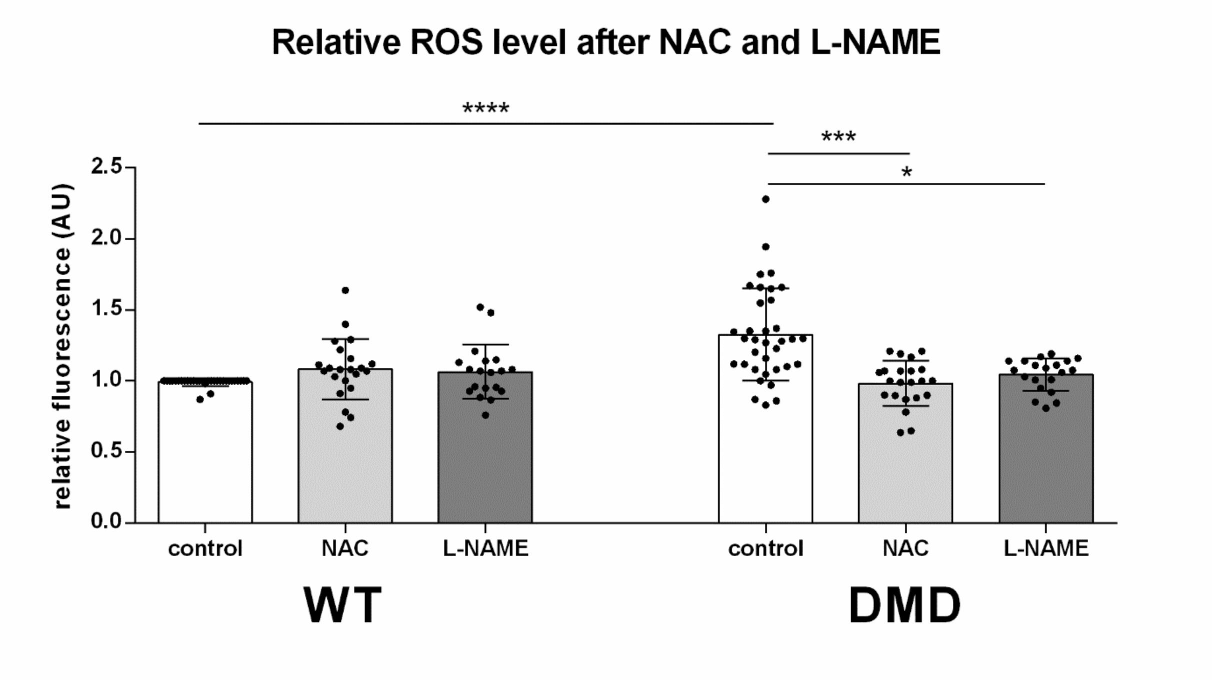

Fig. S8: The spontaneous level of ROS in DMD hPSC lines was significantly elevated compared to the WT hPSC. Graph shows normalized data comparison between pooled data from tested WT hPSC lines and pooled DMD hPSC lines. NAC treatment significantly decreased ROS production in DMD hPSC lines to the level of WT hPSC. Similar effect is shown with L-NAME treatment. Statistical difference was evaluated by Wilcoxon Signed Rank test (*:p<0.05, **:p<0.01,***:p<0.001, ****:p<0.0001). The error bars show standard deviation.

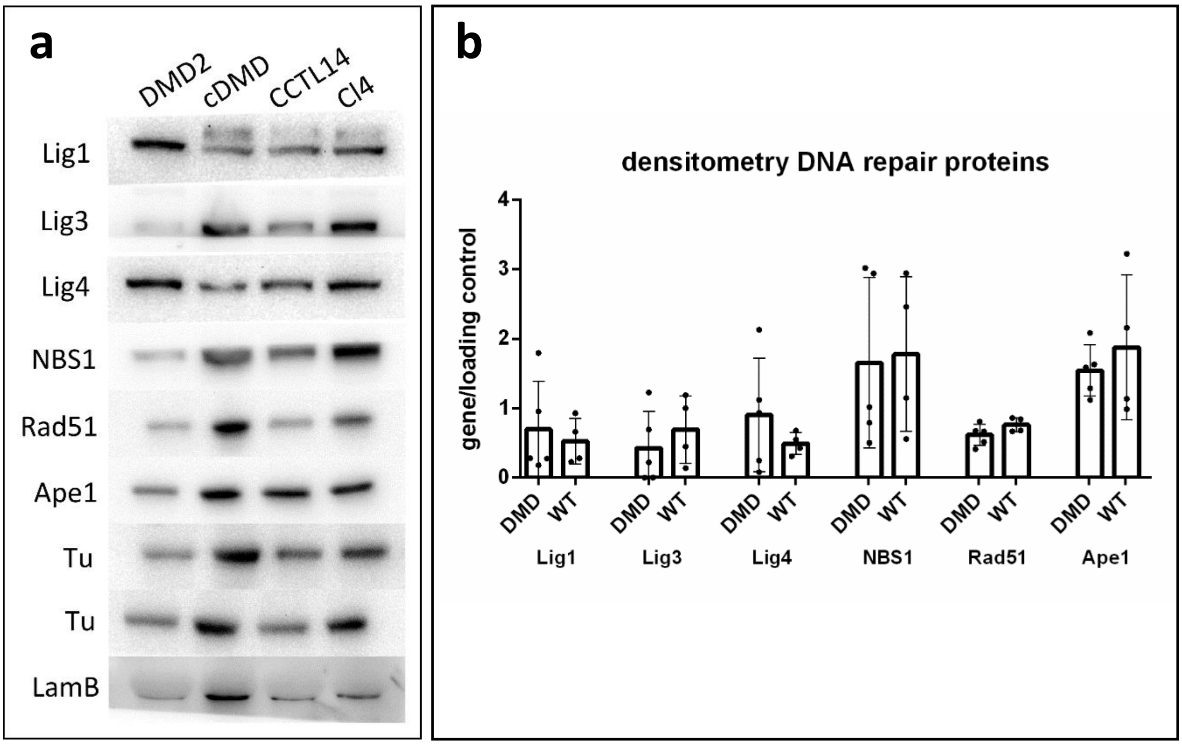

Fig. S9: Protein expression of DNA repair proteins does not change in DMD hPSC. (a) Western blot analysis of proteins responsible for base excision repair {apurinic/apyrimidinic endonuclease APE1 and ligase 1 (LIG1)}, nonhomologous DNA end joining {ligase 4 (LIG4)}, microhomology mediated DNA end joining {ligase 3 (LIG3)} and homologous recombination (recombinase Rad51 and nibrin NBS1). No DMD mutation specific expression was observed. (b) Densitometry was performed (gene/loading control (tubulin or laminB)) from 2 independent repetitions, all available DMD and WT data were pooled separately and analyzed by Student t-test. No significant change was observed.

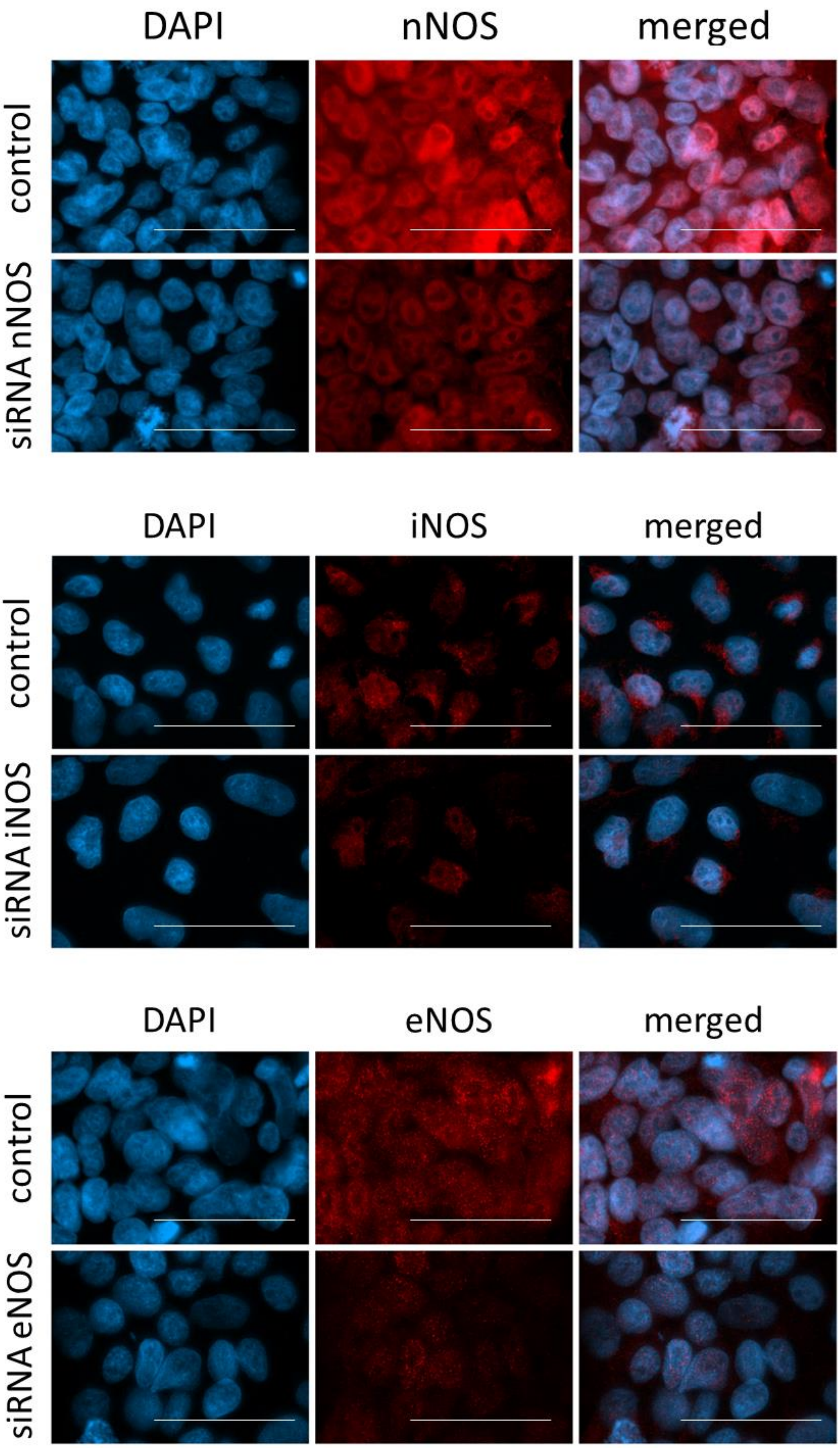
DAPI
nNOS
merged
control
siRNA nNOS
DAPI
iNOS
merged
control
siRNA iNOS
DAPI
eNOS
merged
control
siRNA eNOS

Fig.

S10: Expression downregulation efficiency of iNOS, nNOS and eNOS using siRNA. All silencing experiments were performed with immunocytological control of expression decrease using corresponding NOS isoforms' specific antibodies. Untreated sample was used for comparison (control). Images were acquired with same parameters of acquisition. Line represents 50 μm, NOS isoforms are in red, nuclei are counterstained with DAPI (blue).

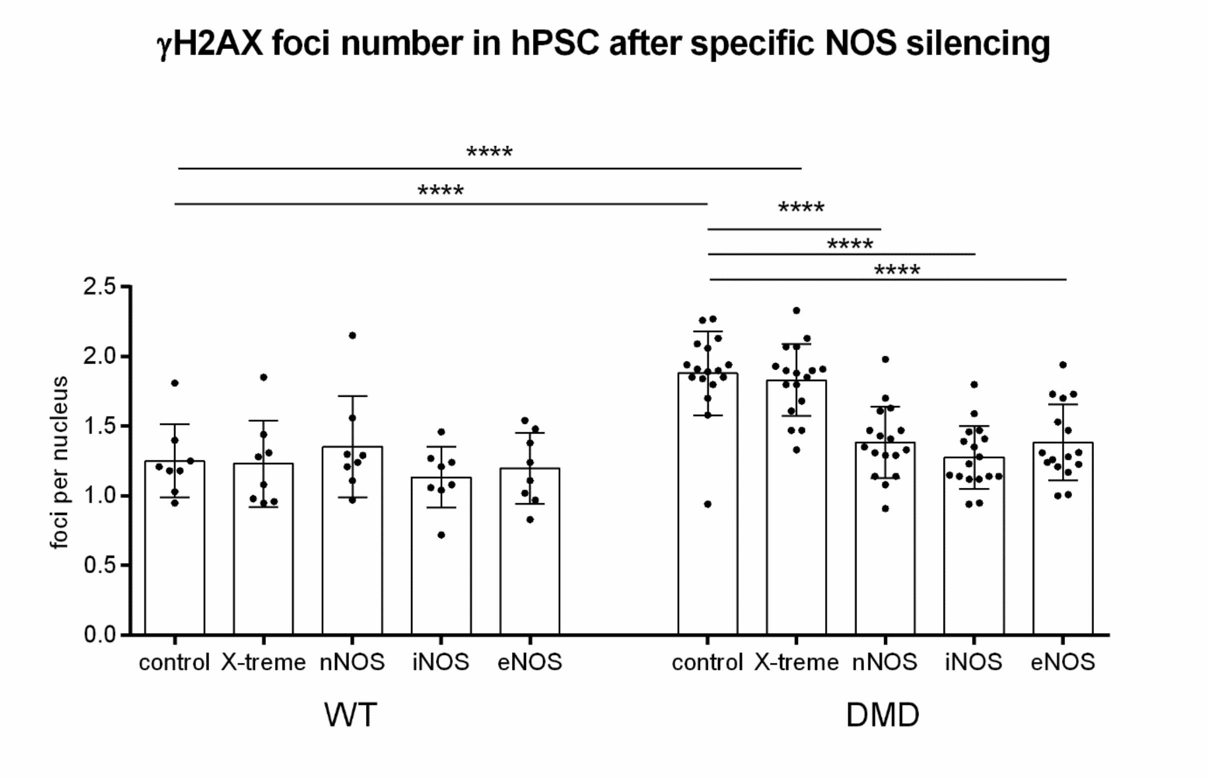

Fig. S11: Individual downregulation if iNOS, nNOS and eNOS using siRNA leads to decreased DNA damage. DNA damage analysis show significant decrease in γH2AX foci formation in DMD hPSC while no significant effect was observed in WT hPSC. Graph shows pooled data from WT hESC and WT hiPSC (WT) and all 3 DMD hPSC lines (DMD02, DMD03, cDMD). The γH2AX formation is significantly decreased in DMD hPSC but not WT hPSC after nNOS, iNOS and eNOS downregulation. Untreated samples (control), samples treated only with transfection reagent (X-treme) and samples treated by individual isoforms' siRNA (nNOS, iNOS and eNOS) are shown. Statistical difference was evaluated by two-way ANOVA and Tukey's multiple comparison test (****:$p<0.0001$). The error bars represent standard deviation.

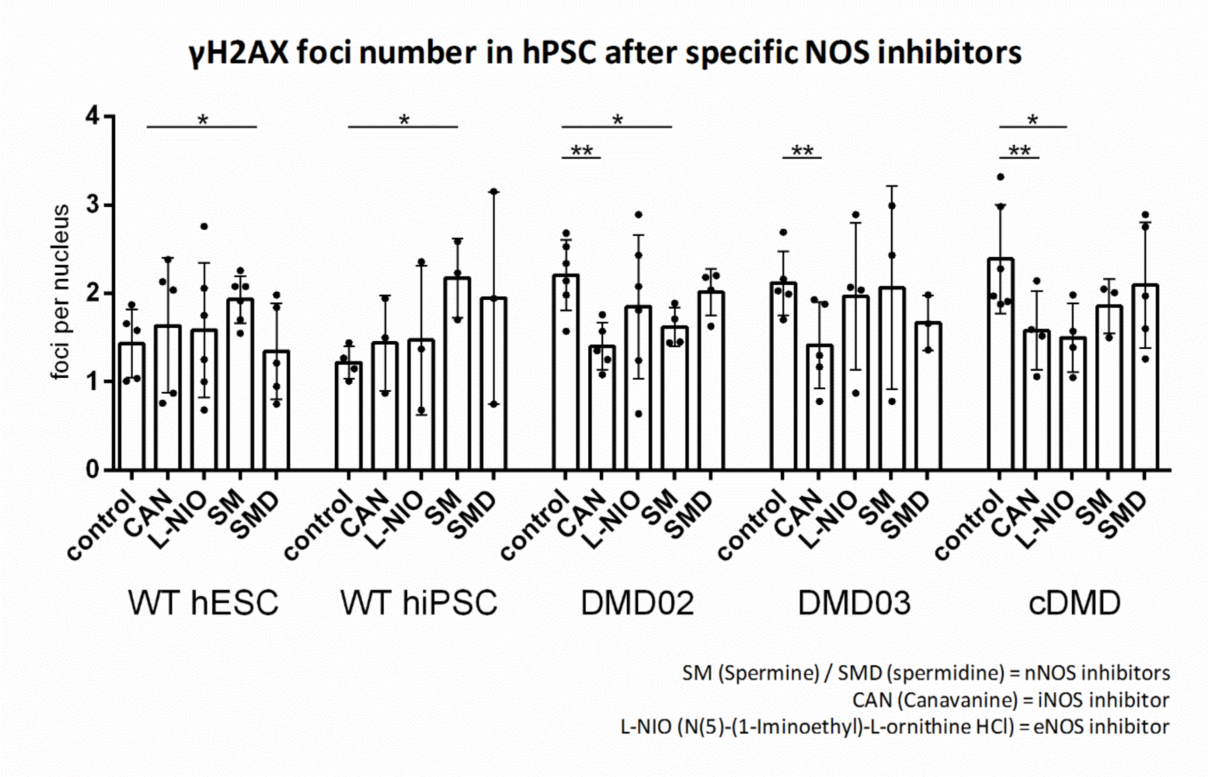

Fig S12: Use of nNOS, iNOS and eNOS specific inhibitors decreases DNA damage in DMD hPSC only partially. DMD hPSC show decreasing trend in γH2AX foci formation in presence of all individual inhibitors with a significant response to CAN in all three DMD hPSC lines, SM in DMD02 and L-NIO in cDMD. No change in DNA damage was observed in WT hPSC with exception of SM that triggered increased γH2AX foci formation in both tested WT hPSC lines. Graph shows data from individual WT hESC and hiPSC and all 3 DMD hPSC lines (DMD02, DMD03 and cDMD). Statistical difference was evaluated by unpaired Student t-test comparing the NOS inhibitors treated (CAN, L-NIO, SM, SMD) cells to their corresponding untreated counterparts (control); *:$p<0.05$**:$p<0.01$, the error bars represent standard deviation.

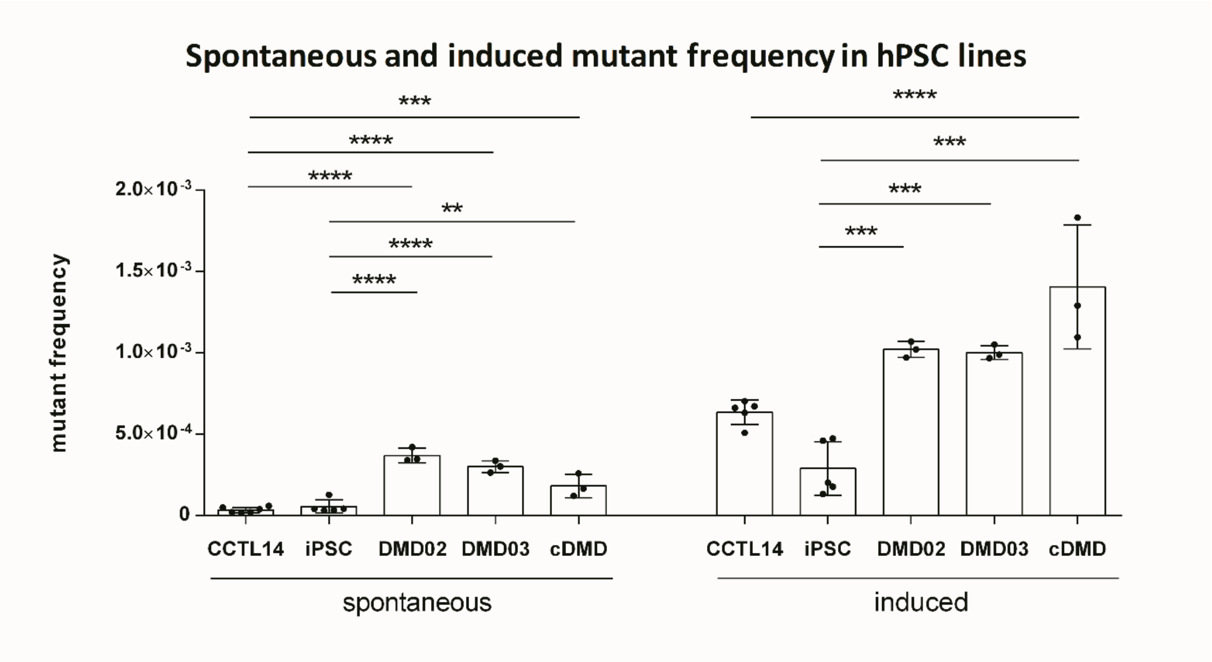

Fig. S13: <u>Spontaneous and IR induced mutation frequency (MF) is elevated in DMD hPSC.</u> HPRT MF assay showed elevated spontaneous MF in both patient specific DMD hiPSC lines and cDMD line compared to WT hESC and WT hiPSC line. The induction of DNA damage with 3Gy of IR resulted in increase in MF in all tested samples but the ratio between DMD and WT corresponding specimen remained the same suggesting multiplicative effect of NOS induced ROS and irradiation induced DNA damage on the overall mutagenesis in DMD hPSC. The values are average of at least three independent biological replicates. The error bars show standard deviation, statistical difference was evaluated by one-way ANOVA and Sidak's multiple comparison test (*:p<0.05, **:p<0.01,***:p<0.001, ****:p<0.0001).

Table S1: **Relative fluorescence of ROS analyzed by CellROX labeling and image analysis**

	**control values**			**N-acetyl cysteine**			**L-NAME**		
	Relative fluorescent intensity (AU) mean±sd	P value*	n	Relative fluorescent intensity (AU) mean±sd	P value**	n	Relative fluorescent intensity (AU) mean±sd	P value**	n
**WT hESC**	1	-/-	17	1,07±0,17	0,4356	17	1,12±0,18	0,1712	15
**WT iPSC**	0,98±0,04	ns/-	5	111±0,34	0,3496	5	0,87±0,08	0,4785	4
**DMD02**	1,38±0,31	<0,001/<0,001	6	0,89±0,18	<0,0001	5	1,02±0,14	0,0025	4
**DMD03**	1,34±0,32	<0,05/<0,01	10	0,92±0,18	0,0001	7	1,01±14	0,0029	7
**cDMD**	1,32±0,32	<0,01/<0,01	8	1,1±0,13	0,0144	8	1,09±0,06	0,0193	8

*calculated by two-way ANOVA and Tukey's post hoc test comparing control values of each line to WT hESC/WT iPSC
**calculated by two-way ANOVA and Dunett's post hoc test comparing the values of NAC and L-NAME treated samples to the untreated controls

Table S2: **Pooled data for WT and DMD hPSC with relative fluorescence by CellROX labeling and image analysis**

	**control values**			**N-acetyl cysteine**			**L-NAME**		
	Relative fluorescent intensity (%) mean±sd	P value*	n	Relative fluorescent intensity (%) mean±sd*	P value**	n	Relative fluorescent intensity (%) mean±sd**	P value**	n
**WT hPSC**	99±3	0,25	31	108±21	0,0558	22	106±19	0,1926	19
**DMD hPSC**	133±32	<0,0001	35	98±16	0,6270/<0,0001	22	104±12	0,1207/<0,0001	20

*calculated by nonparametric Wilcoxon test, comparing DMD hPSC to WT hPSC
**calculated by nonparametric Wilcoxon test comparing each treatment to appropriate untreated control

Table S3: **Exact values of γH2AX foci per nuclei with N-acetyl cysteine and L-NAME treatment**

	**control values**			**N-acetyl cysteine**			**L-NAME**		
	γH2AX mean±sd	P value*	n	γH2AX mean±sd	P value**	n	γH2AX mean±sd	P value**	n
**WT hESC**	1,1±0,3	-/-	6	1,1±0,5	0,66	6	1,1±0,3	0,91	6
**WT iPSC**	1,0±0,2	ns/-	4	1,1±0,2	0,093	4	1,2±0,3	0,0582	4

**DMD02**	2,4±0,5	<0,0001/ <0,0001	6	1,5±0,5	0,0151	6	1,8±0,3	0,02	6
**DMD03**	1,8±0,5	<0,05/ <0,05	7	1,0±0,4	0,0054	7	1,2±0,4	0,0205	7
**cDMD**	2,3±0,5	<0,01/ <0,01	5	1,6±0,2	0,0300	5	1,5±0,2	0,0104	5

*calculated by one-way ANOVA with Tukey's post hoc test comparing control values of each line to WT hESC/WT iPSC

**calculated by unpaired Student t test comparing each treatment to appropriate untreated control

Table S4: **Quantification of NOS isoforms expression**

**line**	**ΔCt nNOS/GAPDH**	**ΔCt eNOS/GAPDH**	**ΔCt iNOS/GAPDH**
**DMD02**	0,0000407	0,000000184911	0,000723192
**DMD02**	0,000583	0,00000302074	0,010308656
**DMD03**	0,000741808	0,000002648	0,001864925
**DMD03**	0,000000320094	0,00000067904	0,001231814
**cDMD**	0,000618045	0,00000189417	0,000602535
**cDMD**	0,000129928	0,000000168587	
**cDMD**	0,0000584		0,00015996
**cDMD**		0,00000513929	0,0000499208
**CCTL14**			0,0000765449
**CCTL12**	0,000194672	0,00000159464	0,000528796
**AM13**	0,0000434586	0,000005339	0,001199515
**cl.4**	0,00000151212	0,00000100109	0,0000144357

Table S5: **Exact values of γH2AX foci per nuclei after specific NOS silencing**

	**control values**			**X-treme**			**nNOS silencing**			**iNOS silencing**			**eNOS silencing**		
	γH2AX mean±sd	*P value**	*n*	*γH2AX mean±sd*	*P value***	*n*	*γH2AX mean±sd*	*P value***	*n*	*γH2AX mean±sd*	*P value***	*n*	*γH2AX mean±sd*	*P value***	*n*
WT hESC	1,2±0,2	-/-	4	1,2±0,2	0,7934	4	1,3±0,2	0,3396	4	1,0±0,2	0,2070	4	1,1±0,2	0,7988	4

WT iPSC	1,4±0,3	ns/-	4	1,3±0,4	0,9520	4	1,4±0,5	0,8716	4	1,3±0,1	0,6880	4	1,3±0,3	0,6967	4
DMD02	2,0±0,1	<0.0010/0,01	3	2,0±0,1	>0,9999	3	1,2±0,3	0,0082	3	1,3±0,2	0,0031	3	1,5±0,2	0,0200	3
DMD03	2,0±0,2	<0.0001/<0,01	6	1,9±0,2	0,5500	6	1,5±0,3	0,0046	6	1,3±0,2	<0,0001	6	1,2±0,1	<0,0001	5
cDMD	1,8±0,2	<0.0100/<0,05	5	1,6±0,2	0,1493	5	1,4±0,2	0,0033	5	1,1±0,2	0,0005	5	1,3±0,3	0,0139	5

*calculated by one-way ANOVA with Tukey's post hoc test comparing each line to WT hESC/WT iPSC

**calculated by unpaired Student t test comparing each treatment to appropriate untreated control

Table S6: **Exact values of γH2AX foci per nuclei after specific NOS silencing, pooled data for WT and DMD hPSC**

	**control values**		**X-treme**		**nNOS silencing**		**iNOS silencing**		**eNOS silencing**	
	γH2AX mean±sd	*P value**	*γH2AX mean±sd*	*P value**	*γH2AX mean±sd*	*P value**	*γH2AX mean±sd*	*P value**	*γH2AX mean±sd*	*P value**
WT hPSC*	1,3±0,3	-	1,8±0,3	ns	1,4±0,4	ns	1,1±0,2	ns	1,2±0,3	ns
DMD hPSC*	1,9±0,3	<0,0001	1,8±0,3	ns	1,4±0,3	<0,0001	1,3±0,3	<0,0001	1,4±0,3	<0,0001

*calculated by two-way ANOVA with Tukey's post hoc test, mutation presence and silencing application provided as evaluation criteria

Table S7: **Pooled data for WT and DMD hPSC with γH2AX foci per nuclei with specific NOS inhibitors**

	**control values**		**canavanine**		**L-NIO**		**spermine**		**spermidine**	
	γH2AX mean±sd	*P value**	*γH2AX mean±sd*	*P value***	*γH2AX mean±sd*	*P value***	*γH2AX mean±sd*	*P value***	*γH2AX mean±sd*	*P value***
WT hPSC*	1,4±0,3	-	1,7±0,6	ns	1,7±0,7	ns	2,1±0,3	ns	1,7±0,8	ns
DMD hPSC*	2,2±0,5	<0,01	1,5±0,4	<0,01	1,8±0,7	ns	1,8±0,6	ns	2,0±0,5	ns

*calculated by two-way ANOVA with Tukey's post hoc test, mutation presence and treatment presence as evaluation criteria

Table S8: **Exact values of γH2AX foci per nuclei with specific NOS inhibitors**

	**control values**			**canavanine**			**L-NIO**			**spermine**			**spermidine**		
	γH2AX mean±sd	P value*	n	γH2AX mean±sd	P value**	n	γH2AX mean±sd	P value**	n	γH2AX mean±sd	P value**	n	γH2AX mean±sd	P value**	n
**WT hESC**	1,5±0,4	-/-	4	1,8±0,7	0,4996	4	1,8±0,7	0,5746	5	2,0±0,3	0,0707	5	1,5±0,5	0,8933	5

**WT hiPSC**	1,2±0,2	0,1587/-	4	1,4±0,5	0,5879	3	1,5±0,9	0,6710	3	2,2±0,5	0,0314	3	2,0±1,2	0,3721	3
**DMD02**	2,2±0,4	0,0272/0,0019	6	1,4±0,2	0,0041	5	1,8±0,8	0,3562	6	1,6±0,2	0,0303	4	2,0±0,3	0,4235	4
**DMD03**	2,1±0,4	0,0479/0,0029	5	1,4±0,5	0,0328	5	2,0±0,8	0,7306	4	2,1±1,1	0,9318	3	1,7±0,3	0,1274	3
**cDMD**	2,4±0,6	0,03881/0,0066	6	1,6±0,4	0,0536	4	1,5±0,4	0,0339	4	1,9±0,3	0,2065	3	2,1±0,7	0,4773	5

*calculated by unpaired Student t test comparing each line to WT hESC/WT hiPSC
**calculated by unpaired Student t test comparing each treatment to appropriate untreated control

Table S9: **Mean values of mutation frequencies in separate lines**

**line**	**Spontaneous MF (mean±sd)**	**Induced MF (mean±sd)**
**WT hESC**	(0,323±0,02)*10^{-4}	(2,9±0,16)*10^{-4}
**WT iPSC**	(0,535±0,90)*10^{-4}	(6,3±0,75)*10^{-4}
**DMD02**	(3,68±0,04)*10^{-4}	[(10,2±0,49)*10^{-4}
**DMD03**	(2,99±0,04)*10^{-4}	(10,0±0,43)*10^{-4}
**cDMD**	(1,80±0,71)*10^{-4}	(14,1±0,38)*10^{-4}
**WT fibroblasts**	(1,55±0,19)*10^{-4}	-
**DMD02 fibroblasts**	(1,64±0,14)*10^{-4}	-
**DMD03 fibroblasts**	(1,74±0,24)*10^{-4}	-

Table S10.: **Antibodies**

**Antigen** (dilution)	**distributor**	identifier
**Nanog (1:200)**	SantaCruz	Cat# sc-33759
**Oct 4 (1:150)**	SantaCruz	Cat# sc-5279
**γH2AX (1:1000)**	BioLegend (San Diego, CA, USA)	Cat# 613402
**Tubulin (1:1000)**	Exbio (Prague, Czech Republic)	Cat# 11-250-C100
**TRA1-81 (1:100)**	Millipore (Burlington, MA, USA)	Cat# MAB4381
**SSEA4 (1:100)**	Millipore	Cat# MAB4304
**dystrophin N-ter (1:50 ICC/1:50-1:300 WB)**	Leica Biosystems (Wetzlar, Germany)	Cat# NCL-DYSB

**nNOS (1:400)**	Thermo Fisher (Watham, MA, USA)	Cat# PA1-033
**iNOS (1:400)**	Thermo Fisher	Cat# PA1-036
**eNOS (1:200)**	Thermo Fisher	Cat# PA5-16887
**laminB (10:100 ICC/1:1000 WB)**	SantaCruz	Cat# sc-6217
**dystrophin C-ter (10:100 ICC/1:1000 WB)**	abcam	Cat# ab15277
**p95/NBS1 (D6J5I) (1:1000)**	Cell Signaling	Cat# 14956
**Rad51 (1:1000)**	Santa Cruz	Cat# sc-8349
**APE1 (1:1000)**	Novus Biologicals	Cat# NB-100-101
**Ligase 1 (1:1000)**	Sigma Aldrich	Cat# HPA048071
**Ligase 3 (1:1000)**	Sigma Aldrich	Cat# SAB2700263
**Ligase 4 (1:1000)**	Sigma Aldrich	Cat# AV34122
**Anti-rabbit AlexaFluor594 (1:500)**	Invitrogen (Carslbad, CA, USA)	Cat# A21207
**Anti-goat AlexaFluor488 (1:500)**	Invitrogen	Cat# A11055
**Anti-mouse IgG HRP conjugated (1:5000)**	Beckman Coulter	Cat# IM0817
**Anti-mouse AlexaFluor488 (1:500)**	Invitrogen	Cat# A11001
**Anti-mouse AlexaFluor594(1:500)**	Invitrogen	Cat# A11005

Table S11: **PCR primers and cycling conditions**

**gene**	**Primer sequence (5′–3′)**	**Annealing temperature (°C)/number of cycles/annealing time (s)**	**Product size (base pairs)**
**AFP**	F: AGA ACC TGT CAC AAG CTG TG	55/30/45	680
	R: GAC AGC AAG CTG AGG ATG TC		
**GATA4**	F: CTC CTT CAG GCA GTG AGA GC	55/30/45	577
	R: GAG ATG CAG TGT GCT CGT GC		
**GATA6**	F: GCC TCA CTC CAC TCG TGT CT	55/30/45	541
	R: TCA GAT CAG CCA CAC AAT ATG A		
**GAPDH**	F: AGC CAC ATC GCT CAG ACA CC	62/22/30	302
	R: GTA CTC AGC GCC AGC ATC G		
**NANOG**	F: CCT ATG CCT GTG ATT TGT GG	62/28/30	208
	R: CTG GGA CCT TGT CTT CCT TT		
**NEUROD 1**	F: GAG TGT CTC AGT TCT CAG GAC G	62/35/30	475
	R: TTG GTG GTG GGT TGG GAT AAG C		
**PAX6**	F: GGC AGG TAT TAC GAG ACT GG	55/30/30	427
	R: CCT CAT CTG AAT CTT CTC CG		
**POU5F1**	1 F: GCA AAG CAG AAA CCC TCG T	62/25/20	168
	R: ACA CTC GGA CCA CAT CCT TC		
**SOX2**	F: ATG CAC CGC TAC GAC GTG A	55/30/30	450
	R: CTT TTG CAC CCC TCC CAT TT		

**VIM**	F: GAC ACT ATT GGC CGC CTG CGG ATG AG	55/25/30	420
	R: CTG CAG AAA GGC ACT TGA AAG C		
**DMD ex 52-54**	F:AGG ATT TGG AAC AGA GGC GTC	64/35/45	351
	R:GTC TGC CAC TGG CGG AGG TC		
**NOX2**	F: CAA GAT GCG TGG AAA CTA CCT AAG AT	63/30/30	102
	R: TCC CTG CTC CCA CTA ACA TCA		
**nNOS**	F: TAG CTT CCA GAG TGA CAA AGT GAC C	62/33/30	220
	R: TGT TCC AGG GAT CAG GCT GGT ATT C		
**iNOS**	F: CAG TAC GTT TGG CAA TGG AGA CTG C	64/28/45	365
	R: GGT CAC ATT GGA GGT GTA GAG CTT G		
**eNOS**	F: CAG TGT CCA ACA TGC TGC TGG AAA TTG	63/30/45	485
	R: TAA AGG TCT TCT TCC TGG TGA TGC C		
**GPX1**	F: GCA ACC AGT TTG GGC ATC AG	67/30/45	141
	R: CGT TCA CCT CGC ACT TCT CG		
**GPX3**	F: CAT CCC CTT CAA GCA GTA TGC	67/30/45	136
	R: GCC CGT CAG GCC CTC AGT AG		
**GPX4**	F: TGG GAA ATG CCA TCA AGT GG	67/30/45	108
	R: GGT CCT TCT CTA TCA CCA GGG G		
**SOD1**	F: TGA AGG TGT GGG GAA GCA TT	67/30/45	204
	R: CAT CGG CCA CAC CAT CTT TG		
**SOD2**	F: CGC TTT CTT AAG GCC CGC	67/30/45	449
	R: GGG TTC TCC ACC ACC GTT AG		
**SOD3**	F: CTC CAA CAG ACA CCC TCC AC	63/35/45	265
	R: AGT CTC AGG GCT TAT GGG GT		
